# Oogenesis and lipid metabolism in the deep-sea sponge *Phakellia ventilabrum* (Linnaeus, 1767)

**DOI:** 10.1038/s41598-022-10058-6

**Published:** 2022-04-15

**Authors:** Vasiliki Koutsouveli, David Balgoma, Antonio Checa, Mikael Hedeland, Ana Riesgo, Paco Cárdenas

**Affiliations:** 1 Department of Life Sciences, The Natural History Museum of London, Cromwell Road, London, SW7 5BD UK; 2 Pharmacognosy, Department of Pharmaceutical Biosciences, Uppsala University, BMC, Husargatan 3, 751 24 Uppsala, Sweden; 3 RD3 Marine Symbioses, GEOMAR Helmholtz Centre for Ocean Research Kiel, Düsternbrooker Weg 20, 24105 Kiel, Germany; 4 Analytical Pharmaceutical Chemistry, Department of Medicinal Chemistry, Uppsala University, BMC, Husargatan 3, 751 23 Uppsala, Sweden; 5 Unidad de Excelencia, Instituto de Biología y Genética Molecular (IBGM), Universidad de Valladolid - Consejo Superior de Investigaciones Científicas (CSIC), Valladolid, Spain; 6 Division of Physiological Chemistry 2, Department of Medical Biochemistry and Biophysics, Karolinska Institutet, 17165 Stockholm, Sweden; 7 Department of Biodiversity and Evolutionary Biology, Museo Nacional de Ciencias Naturales, Calle de José Gutiérrez Abascal, 2, 28006 Madrid, Spain

**Keywords:** Biochemistry, Ecology, Evolution, Molecular biology, Physiology, Ocean sciences

## Abstract

Sponges contain an astounding diversity of lipids that serve in several biological functions, including yolk formation in their oocytes and embryos. The study of lipid metabolism during reproduction can provide information on food-web dynamics and energetic needs of the populations in their habitats, however, there are no studies focusing on the lipid metabolism of sponges during their seasonal reproduction. In this study, we used histology, lipidome profiling (UHPLC-MS), and transcriptomic analysis (RNA-seq) on the deep-sea sponge *Phakellia ventilabrum* (Demospongiae, Bubarida), a key species of North-Atlantic sponge grounds, with the goal to (i) assess the reproductive strategy and seasonality of this species, (ii) examine the relative changes in the lipidome signal and the gene expression patterns of the enzymes participating in lipid metabolism during oogenesis. *Phakellia ventilabrum* is an oviparous and most certainly gonochoristic species, reproducing in May and September in the different studied areas. Half of the specimens were reproducing, generating two to five oocytes per mm^2^. Oocytes accumulated lipid droplets and as oogenesis progressed, the signal of most of the unsaturated and monounsaturated triacylglycerides increased, as well as of a few other phospholipids. In parallel, we detected upregulation of genes in female tissues related to triacylglyceride biosynthesis and others related to fatty acid beta-oxidation. Triacylglycerides are likely the main type of lipid forming the yolk in *P. ventilabrum* since this lipid category has the most marked changes. In parallel, other lipid categories were engaged in fatty acid beta-oxidation to cover the energy requirements of female individuals during oogenesis. In this study, the reproductive activity of the sponge *P. ventilabrum* was studied for the first time uncovering their seasonality and revealing 759 lipids, including 155 triacylglycerides. Our study has ecological and evolutionary implications providing essential information for understanding the molecular basis of reproduction and the origins and formation of lipid yolk in early-branching metazoans.

## Introduction

Lipids are particularly diverse in marine organisms^1,2^. They provide the most important source of energy^3^ and are indicators of the ecosystems’ health^4–7^. Lipids are necessary nutrients^8–11^, but they are also the main structural elements of cell membranes and play critical roles in several physiological functions, e.g. buoyancy^12,13^, tissue growth, immunity, and wound healing^2,3,14^. In addition, there is a direct link between reproduction and lipid metabolism in animals since lipids are used as energy storage^15^ in the eggs during gamete formation and embryogenesis of many marine organisms, including zooplankton, crustacea, cnidaria and fish^16–20^. Nutrient acquisition and transport, lipid metabolism, and yolk accumulation (which dictates the stage of vitellogenesis) are crucial processes during gametogenesis/embryogenesis because they determine the production of a successful gamete and propagule^20–24^, and in parallel they can affect the survival of the adult during reproduction. For this reason, many species synchronize their gametogenesis with periods of high food availability in the environment in order to withstand the high energetic demands^25–27^. Due to its importance, the relationship of lipid metabolism with seasonal environmental changes and the reproductive period has been studied in several marine organisms across their development, addressing questions such as: which types of lipids are preferred for yolk formation; what is the source of these lipids (e.g. to understand the food web dynamics); and what is the timing of lipid synthesis, lipid accumulation and lipid consumption^2,11,16,18,19,28–33^.

Sponges (phylum Porifera), especially within the class Demospongiae, produce an exceptional diversity of lipids^34–40^. As in other animals, lipids are of particular importance in sponges because they participate in several biological processes like cellular signal transduction and cell aggregation^41,42^, adaptation to shifting environmental conditions (temperature, oxygen, osmolarity, nutrients, pressure), defence from predators, and antioxidant activity^38,43–46^. Sponge lipids have also been studied for their potential pharmaceutical applications, e.g. several demosponge fatty acids exhibit antimalarial, antimycobacterial and antifungal activity^1,47^ and many glycolipids have anti-inflammatory, anticomplement, antitumoral, and immunomodulatory properties^48–51^. Regarding their role in reproduction, yolk of lipid origin is also one of the main components, together with protein platelets, of oocytes and embryos of sponges^52^. Sponge yolk has either a homogeneous^53–55^ or heterogeneous structure with a mix of lipids and proteins in the same platelet^56–60^. But notably, in some demosponges, lipid droplets are the only nutrients accumulated within the oocytes^55^. Yolk is formed either by (i) autosynthesis, in which the egg itself produces the nutrients, and (ii) heterosynthesis through phagocytosis of yolk precursors, nutrients and bacteria either provided from accessory cells or taken from the mesohyl of the sponge^60–63^. Currently, the type of sponge yolk is only assessed by electron microscopy observations in which the lipid and protein yolk can be largely recognized by their different electron dense nature^63^.

Almost all sponges have lecithotrophic larvae^52,64^ and only a handful of deep-sea species are known to have direct development (from embryo directly to young sponge without the larval phase)^56,65,66^. With the lack of a planktotrophic phase, all of the nutrients needed for the next developmental stages, until settlement and metamorphosis into a sessile adult sponge, are accumulated in the egg during gametogenesis in the oviparous species or during embryogenesis in the viviparous species. Consequently, the quality and quantity of the yolk is crucial to provide the propagule with all the necessary energy.

Few studies have investigated the variations of sponge lipid metabolism with respect to seasonality. Koopmans et al.^67^ studied fatty acid (FA) composition in North-East Atlantic/Mediterranean Sea demosponge species of the genera *Haliclona* Grant, 1841, *Halichondria* Fleming, 1828 and *Aplysina* Nardo, 1834 and found a strong correlation between the FA composition in the surrounding dissolved organic matter and the sponge FAs during nutrient blooms in spring-summer^67^*.* On the other hand, Lüskow et al.^68^ measuring the lipid content as a fraction of sponge dry weight, found that it remained invariable throughout the year, without being affected by seasonal planktonic blooms or periods of starvation. However, none of these studies linked directly these variations with reproduction. Two other studies, using obsolete methods and rough estimates, have linked the seasonality and nutrient blooms with the physiological status of sponges: Reiswig^69^ quantified the nutritional resources within the sponge tissue and found a direct link to growth and reproduction in the Jamaican population of *Mycale* sp., and Elvin^70^ quantified the tissue growth and reproductive output with respect to various abiotic factors (temperature, light, salinity and nutrient abundance) in *Haliclona* (*Reniera*) cf. *cinerea* (Grant, 1826) (*H*. *permollis* in the publication). On another study, Elvin^71^ investigated the direct relationship of lipid content and reproductive investment in *H*. (*R*.) cf. *cinerea*, finding that both protein and lipid levels increased in female individuals just before the onset of gametogenesis. Similarly, Barthel (1986) found a slight increase in lipid content during the reproduction of *Halichondria panicea* (Pallas, 1766)^72^. However, the nature of the lipids used in reproduction (i) for energetic demands and (ii) for the formation of yolk platelets is still almost completely unknown in sponges. Presently, a single study has investigated the production levels of lysophospholipids, in the homoscleromorph viviparous sponge *Oscarella tuberculata* (Schmidt, 1868), in response to seasonality and reproduction; this study found that their levels increased towards the end of the reproductive cycle: during embryogenesis and larval development^73^.

Understanding the lipid level fluctuations during the reproductive period is fundamental from an ecological point of view, in order to assess nutrient availability, food-web dynamics, and energy requirements of sponge populations. Lipid content dynamics can also inform about the ability to survive and reproduce in case of habitat disturbance, including unbalance in lipid sources due to contamination of the habitat, changes in nutrient blooms, sediment resuspension blocking the filtering system and no access to nutrient uptake and lipid storage. Moreover, such studies are pivotal from an evolutionary point of view, to understand the origin and evolution of yolk formation and lipid composition in Metazoa.

Sponges of the genus *Phakellia* Bowerbank, 1862 (order Bubarida), including *P*. *ventilabrum* (Linnaeus, 1767), are key species in boreal deep-sea sponge grounds of the North Atlantic^74–76^. These habitats, formed by sponges, have an immense ecological importance for the ecosystem due to the crucial role sponges play in biogeochemical cycles^77–82^, and the biodiversity of associated fauna^74,76^. Despite being a very common species, from the British Isles to the coasts of Norway and Sweden, the reproduction of this species has never been investigated. Interestingly, some of its FAs have been previously identified with GC-MS^36,39^. In the present study, we chose to investigate the reproductive strategy of the demosponge *P*. *ventilabrum* in the boreal deep sea with histological observations (light and electron microscopy). Furthermore, we compared the lipid profile (UHPLC-HRMS) and the expression levels (RNA-seq) of genes related to lipid metabolism in reproductive female versus nonreproductive specimens of this species. By doing so, we aimed to determine i) the types of lipids used for yolk formation and ii) the molecular basis of lipid production during oogenesis, which are indicative of the energetic demands during gametogenesis.

## Methods

### Sample collection

Specimens of *Phakellia ventilabrum* were collected in Langenuen and Korsfjord (Western Norway, depth: 95–332 m) in September 2016 and in Kosterfjord (Western coast of Sweden, depth: 89–91 m) at the end of March 2019 (see Table 1). Collections were done with a Remote Operated Vehicle (ROV) (Kosterfjord) and a triangular dredge (Langenuen/Korsfjord). Specimens were identified based on external morphology and spicules by P. Cárdenas and H.T. Rapp (slides for spicule preparations are available upon request to PC and AR). On board, three ~ 5 mm^3^ pieces were cut with sterile and RNAse-free instruments randomly from different parts of the mesohyl of each specimen and fixed in 2.5% glutaraldehyde solution for histological analysis. Another three ~ 5 mm^3^ pieces, from the same specimens, were fixed in RNAlater™ Stabilization Solution (Thermo Fisher Scientific). For lipidomics, a large piece of each specimen was flash frozen in liquid nitrogen, transported in dry ice to Uppsala University and kept at − 80 °C until freeze-drying for the lipidomic analysis.

**Table 1:** Information on the sampling characteristics and the reproductive status of *Phakellia ventilabrum* specimens studied.

Sampling location	Sampling date	Specimens	Status	Code	Number of oocytes/mm^2^	Diameter of oocytes (μm)	Area occupied by oocytes/μm^2^
Skorpeodden, Korsfjord, Norway (59°58.8790′N, 05°22.4371′E)	8-Sep-16	Specimen_1	Oogenesis	Pre-vitellogenic (PV)	5	25 ± 3	0.004415
Langenuen, Norway (59°58.8790′N, 05°22.4371′E)	9-Sep-16	Specimen_2	Oogenesis	Pre-vitellogenic (PV)	2	17 ± 7	0.003803
Langenuen, Norway	9-Sep-16	Specimen_3	Oogenesis	pre-vitellogenic (PV)	3	23 ± 2	0.007567
Skorpeodden, Korsfjord, Norway	8-Sep-16	Specimen_4*	Oogenesis	Vitellogenic_I (Vi_I)	3	40 ± 1	0.047715
Langenuen, Norway	9-Sep-16	Specimen_5	Oogenesis	Vitellogenic_II (Vi_II)	2	71 ± 14	0.075556
Langenuen, Norway	9-Sep-16	Specimen_6*	Oogenesis	Vitellogenic_II (Vi_II)	5	62 ± 2	0.126028
Skorpeodden, Korsfjord, Norway	8-Sep-16	Specimen_7	Non-reproductive	–		–	–
Skorpeodden, Korsfjord, Norway	8-Sep-16	Specimen_8*	Non-reproductive	–		–	–
Skorpeodden, Korsfjord, Norway	8-Sep-16	Specimen_9*	Non-reproductive	–		–	–
Krugglöbranten, Sweden (58°53.10′N, 11°06.04′E)	28-Mar-19	Specimen_10	Oogenesis	Vitellogenic_II (Vi_II)	2	57 ± 14	0.039863
Krugglöbranten, Sweden	29-Mar-19	Specimen_11	Oogenesis	Vitellogenic_II (Vi_II)	2	53 ± 11	0.028747
Krugglöbranten, Sweden	28-Mar-19	Specimen_12	Non-reproductive	–		–	–
Krugglöbranten, Sweden	29-Mar-19	Specimen_13	Non-reproductive	–		–	–

The asterisks signal the samples that were also used for the transcriptomic analysis.

### Histological analysis

Sample preparation for histological analyses was done according to the protocol by Koutsouveli et al.^83^. In brief, the fresh collected samples, once in the lab, were first rinsed in a solution of 0.6 M NaCl and 0.4 M PBS and then post fixed in 2% osmium tetroxide in 0.4 M PBS for two hours and incubated overnight in 4% hydrofluoric acid (HF) to remove any silica structures from their skeleton. Afterwards, samples were rinsed with distilled water and dehydrated with ethanol in an ascending series (50–70–96–100%). For light microscopy, samples were then embedded in paraffin blocks and those were cut with a HM325 microtome (ThermoFisher Scientific) into sections of 5 μm, which were stained with a standard Harris’ Hematoxylin and Eosin (HandE) protocol. Histological sections were observed with an Olympus microscope (BX43) with an attached UC50 camera. For transmission electron microscopy (TEM), samples were embedded in LRW resin blocks (agar Scientific) (according to the guidelines of the manufacturer) and ultrathin sections (60 nm) were cut with an Ultracut Reichert-Jung ultramicrotome. Then, sections were stained with 2% uranyl acetate/lead citrate^84^, and observed with a Hitachi TEM microscope (H-7650) at 80 kV.

We then measured the size and number of gametes of the reproductive females on the histological sections, with the Olympus Microimaging software CellSens standard integrated to the Olympus microscope. Several tissue areas (0.58 mm^2^ each area) of each section were surveyed for the measurements, avoiding to outnumber the density of the gametes within the tissue. All the images for measurements were taken at 10 × magnification. To extract the final measurements, we calculated the average and the standard deviation of the size of the gametes from all the different images. We also conducted quantification of the different types of yolk content within the oocytes with ImageJ^85^ on the TEM images. The different types of yolk can be discriminated: protein yolk is highly electrodense (dark/black) in the TEM image while the lipid yolk is less dense (light grey).

Information extracted from the histological observations of the reproductive specimens, regarding the developmental stage of their oocytes, was further used for the lipidomic and transcriptomic analyses. Specifically, for the lipidomic analysis the area of sponge tissue occupied by oocytes was calculated in order to observe the variation of lipid signal. As the number of oocytes did not change in the different developmental stages of oogenesis, the increase of sponge tissue occupied by oocytes was proportional to the maturation stage of the oocytes. For transcriptomic analysis, individuals with reproductive elements were classified as Vitellogenic_I (Vi_I) or Vitellogenic_II (Vi_II) stages, based on the developmental stage of their oocytes observed in the histological sections, while nonreproductive specimens (NR) did not have any gametes. Vi_II stages contain the most mature oocytes, which are larger in size and have greater yolk content than the Vi_I individuals.

### Lipidomic analysis

#### Semi-targeted lipidomics

The samples from the different locations (Langenuen/Korsfjord and Kosterfjord) were processed in different batches. Frozen subsamples were freeze-dried and grinded in a falcon tube; 52 ± 2 mg and 22 ± 1.2 mg of powder was extracted for the samples of Langenuen/Korsfjord and Kosterfjord respectively (Supplementary Table S9A). The extraction was done with chloroform, based on the Bligh and Dyer protocol^86^. Briefly, we incubated the samples in 1:2 chloroform/methanol (v/v) overnight. The organic phase (lower phase) was collected after centrifugation, mixed with 375 μL of CHCl_3_ and incubated for another 24 h. The organic phases from the two extractions were pooled (1 mL) together and dried under vacuum. The samples were resuspended in 200 µL of ACN/IPA 50:50. We also prepared the quality control (QC) in which we mixed 10 µL from all the extracted specimens. The separation was slightly modified from Balgoma et al.^87^. Briefly, lipids were separated by Ultra High-Performance Liquid Chromatography (UHPLC) with a BEH C18 column (1.7 µm, 2.1 × 150 mm) on an Acquity chromatographer hyphenated to a Synapt G2S QToF (Waters, Manchester UK). The mobile phases were A) water/acetonitrile/isopropanol 40:30:30 (v/v/v) with 5 mM of ammonium formate, and B) acetonitrile/isopropanol 40:60 (v/v) with 5 mM of ammonium formate. The gradient (flow 0.275 mL min^-1^) changed linearly as in Supplementary Table S9B. The ionization was carried out by electrospray in positive and negative modes. The injections of QC were four at the beginning and at the end of the injections as well as between every five injections of the samples. During the extraction protocol and injection, the order of the samples was randomized.

#### Oxylipin quantification

For the extraction of oxylipins we followed the protocol by Kolmert et al.^88^. In detail, 30 ± 6 mg of tissue for each specimen (Supplementary Table S9A) were mixed with 2 mL of methanol and sonicated for 30 min on ice to enhance the extraction. After centrifugation (15 min at 3000*g*), the supernatant (1.5 mL) was transferred into Pyrex extraction tubes. We evaporated the solvent with nitrogen (TurboVap LV) until reaching an approximate volume of 300 μL and then we added 2.7 mL of Solid Phase Extraction (SPE) buffer (pH 5.6). SPE was performed in order to eliminate any interferences that could add background signals to our analysis using an Extrahera automated system. After loading the column with the extract, we washed with 2.5 mL of 90:10 H_2_O/MeOH (v/v) and the extract of interest was eluted with 100% MeOH. The eluent was dried under nitrogen (TurboVapLV™ evaporator) and resuspended in 100 µL of 85:15 MeOH/H_2_O (v/v). Finally, the samples were filtered with 0.1 μm nylon filters (Amicon Ultrafree-MC) by centrifuging at 5000*g* for 3.5 min at 4 °C. We collected approximately 80 μL of the final extract and 8 μL were injected on a BEH C18 column (1.7 μm, 2.1 × 150 mm). For chromatographic separation and lipid quantification, we used an Acquity UPLC coupled to Triple Quadrupole Mass Spectrometer, TQS-S™ instrument (Waters, Milford, USA), operated in negative mode. Mobile phases consisted of A, water with 0.1% of acetic acid and B, acetonitrile/isopropanol 90:10. Column temperature was 60 °C and flow rate was 0.5 mL min^−1^ with a gradient initiated at 90% of A that changed linearly to 65% in min 3.5, to 60% in min 5.5, to 58% in min 7, to 50% in min 9 and to 35% in min 15. Further details about the mass spectrometry parameters can be found in Balgoma et al.^89^.

#### Data pre-treatment

For lipidomics data-pretreatment we followed Balgoma et al.^87^. Briefly, Water’s raw mass spectrometry files were transformed with Data Bridge into CDF files and processed with XCMS package in R^90,91^. To identify the lipids, a database of lipids from Lipid Maps was used^92^. For this database, the *m/z* of the different adducts was generated in R by package Rdisop^93^. For every lipid, the adduct, the total number of carbons and the total number of unsaturations were identified from the combination of *m/z* and retention time of the first and the second isotopologues (Supplementary Table S1). When possible, because of the intensity, the fatty acids were identified by the fragmentation patterns (Supplementary Table S1). The lipid signal was quantified as the area under the chromatographic curve of the peaks. For every specimen, the lipid signal was normalized by the weight of sample extracted in mg. This method of lipid signal estimation, is a relative quantification method. Accurate or semi-quantification would need internal standards, e.g. lipids with odd-numbered carbon chains, that could not be used in sponges, as the latter are very rich in odd-numbered fatty acids. Regarding oxylipins, for the quantification, a calibration curve with 11 external points, spiked with internal standards was used^88,89^. Concentration calculations were performed in TargetLynx (Waters, Milford, USA). Finally, the amount of oxylipins were normalized as ng of oxylipin per g of dry sponge extracted.

#### Statistical analysis

First, to study the factor location/month, we used a principal component analysis (PCA) and we applied multiple t-tests for the main lipid categories and subcategories according to the number of saturations in order to observe variations between the two different sampling locations/months. In the case of oxylipins, the data were available only for specimens from one location (Langenuen/Korsfjord) so they were not included in this analysis. Significant variations had an adjusted *p* value (based on Benjamini and Hochberg, BH; FDR) ≤ 0.05.

To isolate the effect of the reproduction status from location, we performed multivariate regression on the logarithm of the signal of the lipids of all the identified lipid categories by using the percentage of the area of sponge tissue occupied by oocytes as predictor. Consequently, the coefficient for the percentage of the area of the sponge tissue occupied by oocytes represented the upregulation (positive coefficient) or downregulation (negative coefficient) of the lipid with the development of oocytes (Supplementary Table S2). From nonreproductive to female individuals with most mature oocytes in their tissue, the area of the tissue occupied by oocytes increased. For the analysis, each lipid category was analysed (Supplementary Table S1; Supplementary Table S2). All the statistical and graphical analyses were conducted in R v3.4.2 (R Core Team 2017).

### Transcriptomic analysis

#### RNA extraction and library preparation

Given logistic and sampling limitations, only four samples of *P. ventilabrum* from Langenuen/Korsfjord were used for the transcriptomic analysis (Table 1, with asterisks): two females and two nonreproductive specimens. The protocols used for RNA isolation, mRNA purification and cDNA library preparation were described in Koutsouveli et al.^94^. Briefly, total RNA extraction was conducted with a standard TRIzol™ Reagent (ThermoFisher Scientific) protocol, according to the guidelines of the manufacturer. Further mRNA purification was performed with the Dynabeads mRNA DIRECT kit (ThermoFisher Scientific), applying the final stage of the protocol, ‘Elimination of rRNA contamination’. The quantity and quality of mRNA were assessed by NanoDrop 2000 (ThermoFisher Scientific). Then, cDNA libraries were prepared with Scriptseq v2 kit (Illumina) (according to the manufacturer’s instructions), using an initial mRNA quantity of 50 ng. The amount of cDNA was then assessed with Qubit™ dsDNA HS Assay kit (ThermoFisher Scientific) and the quality with an Agilent Tapestation 2200 system (Agilent Technologies). The sequencing was done in an Illumina NextSeq 500 platform at the Natural History Museum of London sequencing facility (Molecular Core Labs).

#### Assembly, differential gene expression analysis, annotation

Filtering of reads based on quality was performed with Trimmomatic^95^, and the de novo assembly was done with Trinity v2.8.4^96^. Completeness of the assembly was calculated with Benchmarking Universal Single-Copy Orthologs (Busco V2/3) against metazoan cassettes^97^. For gene expression analyses, mapping of the reads to the reference assembly was performed with Bowtie2^98^, transcript quantification was done with RSEM^99^, and differential gene expression (DGE) analysis was conducted with edgeR^100,101^. We did a pairwise comparison of the two female individuals versus the two nonreproductive specimens and we also did pairwise comparisons between each female specimen and the two nonreproductive specimens because the two female specimens were in different developmental stages (Vi_II containing more mature oocytes than Vi_I), and like this we could obtain more accurate information, regarding their physiological processes. Due to lack of replication for the reproductive condition, we used dispersion 0.1 for the conduction of DGE analysis with edgeR, as indicated by the software.

#### Gene Ontology and KEGG enrichment analysis

For the transcriptome annotation, we did a blastx search^102^ of the transcriptome using the *swissprot* database^103^ containing only metazoan proteins (accessed in 2020), using Diamond^104^ with a cut-off e-value of 1e-5. The sequences with blast hits were further annotated by Blast2GOPRO^105^ to retrieve the functional information from the Gene Ontology (GO) terms.

We then performed a GO enrichment analysis using a Fisher’s Exact Test in Blast2GOPRO^105^ with a *p* value threshold of ≤ 0.05. This analysis was conducted using as “test *dataset*” the upregulated genes of each female developmental stage separately (Vi_I, Vi_II) versus nonreproductive individuals and as “*reference dataset*” the total annotation file of the reference transcriptome. The percentage of sequences contained in each GO term was extracted and used for the depiction of a bubble graph in R (R Core Team, 2018). In some cases, more than one sequence was linked to the same process, so we added the relevant sequences to have a final summary percentage.

In addition, we performed a KEGG analysis^106^ in Blast2GOPRO^105^ in order to see which biochemical pathways were activated and enriched in Vi_I developmental stage and in Vi_II stage separately, when compared to nonreproductive specimens.

## Results

### Reproductive biology

#### Reproductive season

Reproductive activity was found in specimens collected both in March (Swedish coast) and September (Norwegian coast) (Table 1). *Phakellia ventilabrum* is an oviparous species, as no further developmental stage, e.g. embryos, was observed within the tissue. Although no male individuals were found in any of the locations, we expect *P. ventilabrum* to be gonochoristic since all species of Bubarida follow this strategy, but we cannot exclude the possibility of successive hermaphroditism. At the population level, 50% of the collected specimens (two out of four) were female in the Swedish coast in March, while 66.6% (six out of nine) were female in the population of the Norwegian coast, in September (Table 1). There was moderate asynchrony in the development of the gametes within the populations, with some individuals having previtellogenic oocytes, while others had oocytes in a more advanced developmental stage. Asynchronous oocyte development was also observed within the same individual in a few cases. The density of oocytes was around 2 to 5 oocytes per mm^2^ sponge tissue (Table 1).

#### Ultrastructure of oocytes

**Figure 1:**
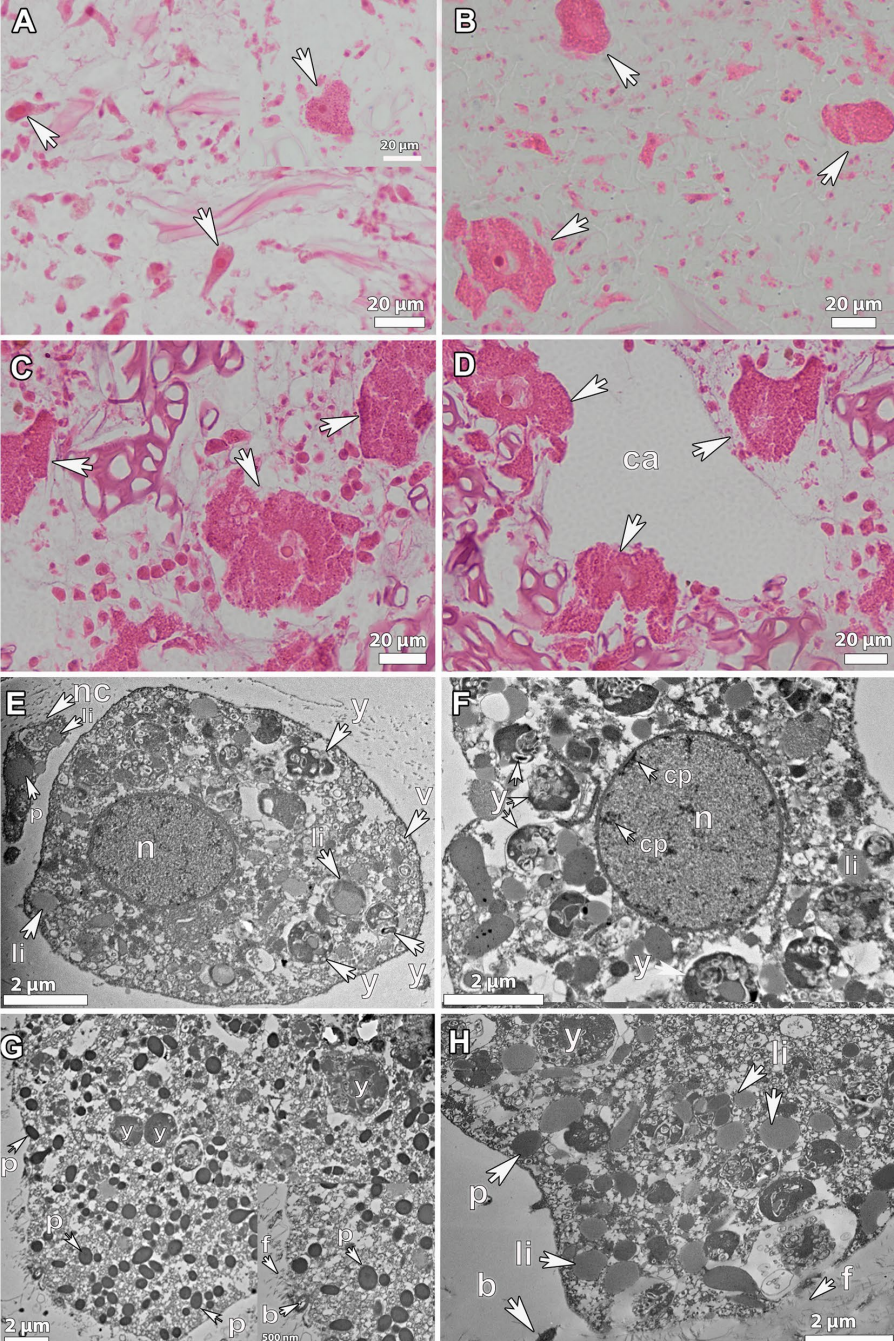
Histological observations of female gametes of *Phakellia ventilabrum*. (**A**) Previtellogenic oocytes (~ 20 μm) distributed in the mesohyl of a female sponge collected in September in Langenuen, Korsfjord. Insert: a close-up of a previtellogenic oocyte with a well-formed nucleus. (**Β**) Oocytes of vitellogenic_stage I (30–40 μm) spread throughout the mesohyl of a female sponge collected in September in Skorpeodden, Korsfjord. (**C**) Oocytes of vitellogenic_stage_II (60–80 μm), from a specimen collected in September in Langenuen, Korsfjord. (**D**) Some of the oocytes from the same specimen were accumulated around the canal (ca), to be released in the water column. (**E**) Ultrastructure of a vitellogenic_stage_I oocyte with a well-formed nucleus (n): the ooplasm contains empty vesicles (v) and large droplets of heterogeneous (y) and lipid (li) yolk. In close proximity, a nurse cell (nc) contains protein (p) and lipid (li) yolk. (**F**) A close-up of the ooplasm from an oocyte of vitellogenic stage I. The ooplasm was full of lipid (li) and heterogenous (y) yolk and the nucleus, (n) contained some chromatin compaction (cp). (**G**) Ultrastructure of a vitellogenic_stage_II oocyte with the ooplasm full of protein (p) yolk and few big droplets of heterogeneous yolk (y). Insert: A fibular structure (f) for support and protection of the oocyte was formed in the surrounding of the oocyte. Note the phagocytosis of a bacterium (b) from the mesohyl. The ooplasm was full of proteic (p) yolk. (**H**) Ultrastructure of a different vitellogenic stage II oocyte: mesoplasm with plenty of lipid (li) yolk and proteic (p) and heterogenous yolk (y). Oocytes are indicated with white arrows in the images (**A**)–(**D**).

**Figure 2:**
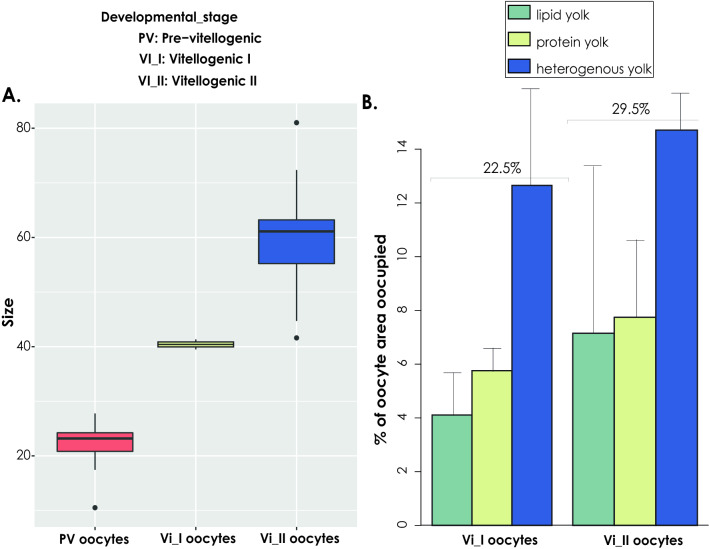
Measurements of oocyte size and yolk content. (**A**) Average size (μm) of previtellogenic (PV), vitellogenic_stage I (Vi_I) and vitellogenic_stage II (Vi_II) oocytes. (**B**) Percentage of the oocyte area occupied by the different types of yolk. For these measurements one specimen with vitellogenic stage I oocytes and another specimen with vitellogenic stage II oocytes were used.

The previtellogenic oocytes of *P*. *ventilabrum*, with an already well-formed nucleolated nucleus, had an average size of 20 ± 5 μm at the beginning of their formation (Figs. 1A, 2A). During their maturation phase, Vi_I, oocytes increased in diameter to ~ 40 μm, (Figs. 1B, 2A), and at a later vitellogenic stage, Vi_II, oocytes reached a diameter of 80 μm (Figs. 1C, 2A). We expect that the maximum size of oocytes would not surpass 100 μm in diameter as Vi_II oocytes were already close to the canals, ready to be released (Fig. 1D). During the maturation phase, nutrients of lipidic or heterogeneous form started to accumulate in the ooplasm (Figs. 1E, 2B). Nurse cells were present in the proximity of the oocytes, since their early formation, and they contained lipidic yolk, indicating that they probably provide the oocyte with already formed yolk and other nutrients (Fig. 1E). All three different types of yolk: lipidic, proteinaceous and heterogenous (mix of lipid and protein) were present in both Vi_I and Vi_II oocytes (Figs. 1E–H, 2B). Between 22.5% and 29.5% of the oocyte area was filled by yolk in the Vi_I and Vi_II stages respectively (Fig. 2B). Heterogenous yolk was the most abundant type of yolk observed in the oocytes (Figs. 1E–H, 2B). At the Vi_I stage, big droplets of heterogeneous yolk were mainly observed (Fig. 1E–F) with only a few homogeneous lipid and protein droplets (Figs. 1E–F, 2B). From Vi_I to Vi_II, all three types of yolk increased both in number and size (Fig. 2B). In some Vi_II oocytes, protein platelets were more abundant (Fig. 1G), while in some others, lipid droplets were equally abundant to the other two types of yolk (Fig. 1H). Altogether, lipid and protein yolk were formed in similar amounts while heterogenous yolk occupied twice the area of the ooplasm, compared to the other two (Fig. 2B). Although vertical transmission of associated microbial symbionts was not observed in this species, phagocytosis of bacteria by the oocyte was observed in a single instance (Fig. 1G, insert). Given our histological observations of several specimens of *P. ventilabrum* (> 20 specimens) from different areas and years, we consider that *P. ventilabrum* is a Low Microbial Abundant (LMA) species, as almost no bacteria were observed in the mesohyl (Fig. 1E,G,H).

### Lipidomic analysis

**Figure 3:**
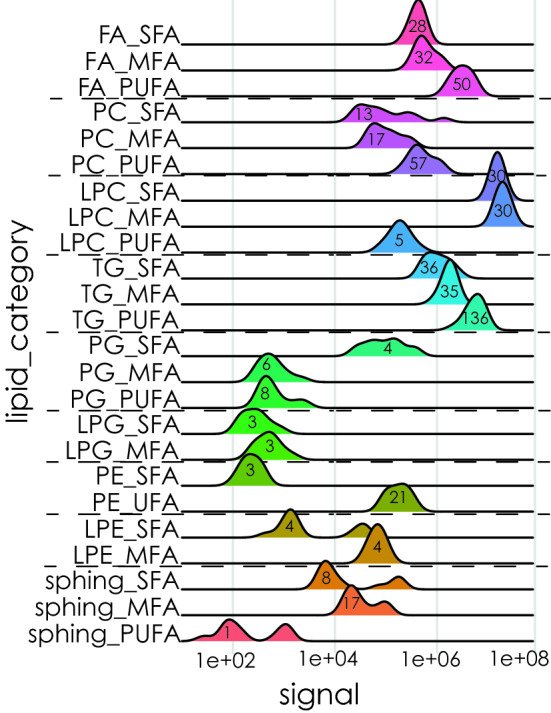
Signal of different lipid categories*.* The log signal of saturated fatty acids (SFA), monounsaturated fatty acids (MFA), or polyunsaturated fatty acids (PUFA), free fatty acids (FFA), phosphatidylcholines (PC), lysophosphatidylcholines (LPC), triacylglycerides (TG), phosphatidylglycerols (PG), lysophosphatidylglycerols (LPG), phosphatidylethanolamines (PE) and lysophosphatidylethanolamines (LPE), sphingolipids and glycosphingolipids. In each peak the number of detected lipids is indicated.

To characterize the changes of the lipidome in relation to the reproductive status, we analysed the samples by semi-targeted mass spectrometry lipidomics with relative quantification. In total, we detected 759 different lipids in the extracts of *P*. *ventilabrum* with a remarkable variety of lipids (Supplementary Table S1). The main lipid categories were: 96 free fatty acids (FFAs) (Supplementary Table S1), 61 phosphatidylcholines (PC) (Supplementary Table S1) and 155 triacylglycerides, (TGs) (Supplementary Table S1); and finally, 26 sphingolipids of ceramide (Cer) and glycosphingolipids of ceramide (Glc-Cer) (Supplementary Table S1). In most of the above lipid categories, the highest number of lipids detected with our analysis was unsaturated fatty acids (UFA) (344 lipids), and most particularly polyunsaturated fatty acids (PUFA) (251 lipids) (Fig. 3), which also had the highest signals within each lipid category (Fig. 3). Exceptions were the phosphatidylglycerols (PGs), lysophosphatidylcholines (LPCs) and sphingolipids for which the number of saturated fatty acids (SFA) or monounsaturated fatty acids (MFA) detected was higher than PUFA (10 SFA/MFA vs 8 PUFA; 60 SFA/MFA vs 5 PUFA; 25 SFA/MFA va 1 PUFA respectively) and had higher signal detections (Fig. 3).

We also identified and determined 62 oxylipins by absolute quantification, derived from polyunsaturated fatty acids (PUFAs) such as arachidonic acid (AA), linoleic acid (LA), α-linolenic acid (ALA), eicosapentaenoic acid (EPA), and docosahexaenoic acid (DHA) and several oxylipins derived from those (Supplementary Table S1). Among others, several prostaglandins (PGs) were identified, i.e. PGD_2_ (prostaglandin D_2_), PGE_2_ (prostaglandin E_2_), PGF_2α_ (prostaglandin F_2_), prostaglandin E_3_ (PGE_3_), and prostaglandin F_3α_ (PGF_3α_) (Supplementary Table S1).

#### Lipid signal variations in different locations/months

**Figure 4:**
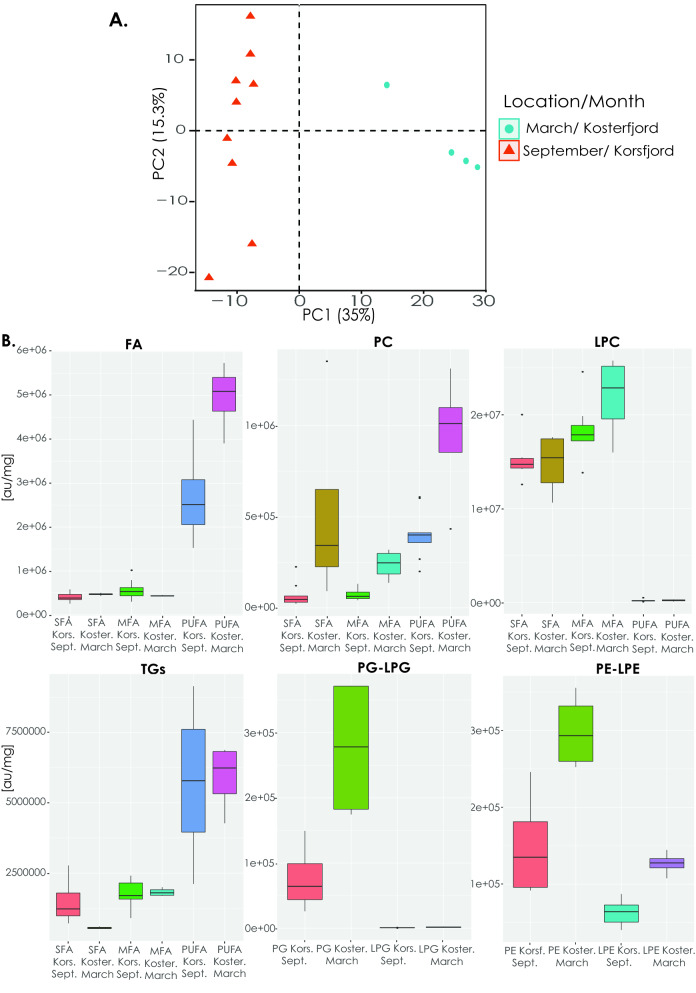
Lipid signal of different lipid categories in different locations/months. (**A**) Principal Component Analysis (PCA) indicating the scores of the first two principal components of the lipidome in the sponge individuals between the two different locations. (**B**) Boxplots depicting the average signal [au/mg] extracted from all the lipids of each lipid category in the two different sampling locations/months. Fluctuations in signal [au/mg] were calculated separately for saturated (SFA), mono-unsaturated (MFA), and poly-unsaturated (PUFA) lipids within each lipid category. Abbreviations: free fatty acids (FFA), phosphatidylcholines (PC), lyso-phosphatidylcholines (LPC), triacylglycerides (TGs), phosphatidylglycerols (PG), lyso-phosphatidylglycerols (LPG), phosphatidylethanolamines (PE), lyso-phosphatidylethanolamines (LPE), saturated (SFA), mono-unsaturated (MFA), poly-unsaturated (PUFA).

The main variation in the lipidome among the specimens was due to the different sampling locations/months or both, independently of the reproductive status (Fig. 4Α). Among the different locations, the total signal of almost all the main lipid categories studied was higher in samples collected in Kosterfjord (Sweden) in March than in Korsfjord (Norway) in September (Fig. 4Β; Supplementary Table S1). In almost all the lipid categories, the PUFA lipids had the most varied signal between the locations/months (PUFA-FFA; PUFA-PC; PUFA-TG; PUFA-PE) (Fig. 4B; Supplementary file SF1).

#### Lipid signal variations dependent on the reproductive status

**Figure 5:**
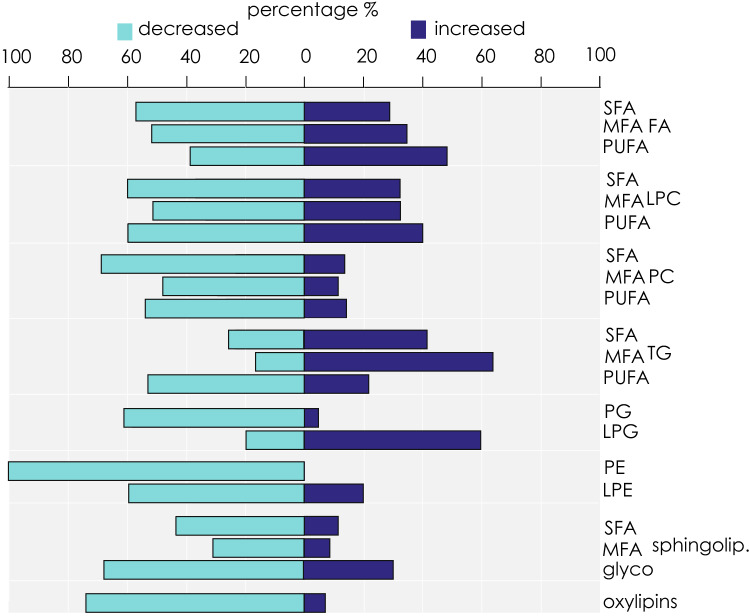
Signal of different lipid cateogires among the different reproductive categories. Barplot presenting the sum of SFA-, MFA-, and PUFA- lipids within each lipid category with a trend of total signal decrease or increase in female, compared to nonreproductive individuals within each lipid category. Abbreviations: free fatty acids (FFA), phosphatidylcholines (PC), lyso-phosphatidylcholines (LPC), triacylglycerides (TGs), phosphatidylglycerols (PG), lyso-phosphatidylglycerols (LPG), phosphatidylethanolamines (PE), lyso-phosphatidylethanolamines (LPE), saturated (SFA), mono-unsaturated (MFA), poly-unsaturated (PUFA).

**Figure 6:**
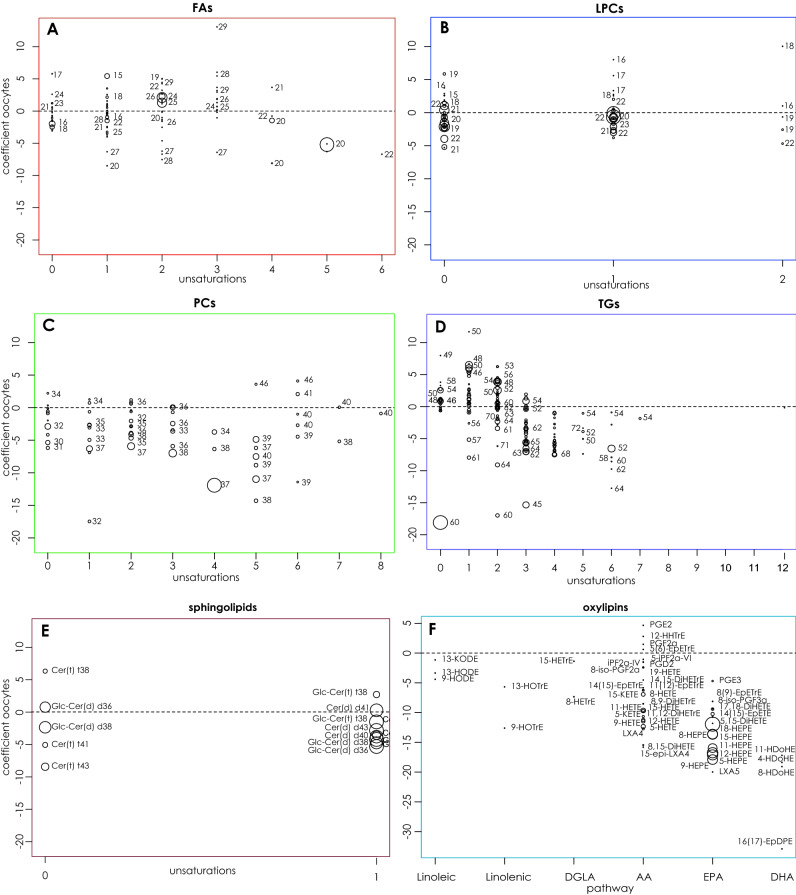
Relationship between lipids and the area of sponge tissue occupied by oocytes for the different lipid categories. (**A**) free fatty acids (FFA); (**B**) lysophosphatidylcholines (LPC); (**C**) phosphatidylcholines (PC); (**D**) triacylglycerides (TGs); (**E**) sphingolipids; (**F**) oxylipins. The graph depicts the coefficient of the regression analysis between the logarithm of the signal of the lipids and the area of the sponge tissue occupied by oocytes (correcting by the location). Values of the regression coefficient above 0 mean upregulation of the lipid signal with the increasing tissue area occupied by oocytes. Similarly, values below 0 mean downregulation of the lipid signal with the increasing tissue area occupied by oocytes. The non-reproductive individuals are included in the analysis as those without oocyte, i.e. area of oocyte = 0. To distinguish major from minor species in the same family, the area of the circles is proportional to the signal of the lipid in the control group. The number next to the circles indicates the number of carbons of the lipid.

Regarding the general signal fluctuation of each lipid category, most lipids indicated a tendency to decrease their signal with the oocyte maturation (increasing area occupied by oocytes), with the exception of the TGs, which in their majority increased their signal with oocyte maturation (Figs. 5, 6; Supplementary Table S2).

Specific observations were made on the lipids of each lipid category, based on their number of carbons and unsaturation. Particularly regarding the FFAs, the signal of a higher percentage of SFAs (57%) and MFAs (56%) decreased (vs 28% and 37% which increased respectively) with oocyte maturation (the area occupied by oocytes increased) (Fig. 5), while an almost equal percentage of PUFAs had their signal decreasing (39%) or increasing (47%) (Fig. 5). Specifically, PUFA FFA with 2 or 3 unsaturations showed an increase, while the signal of those FFAs with a higher number of unsaturations (4, 5, or 6) tended to decrease in relation to the increasing area of the oocytes (Fig. 6A). As for the LPCs, the signal of most SFA (60%), MFA (53%) and PUFA (60%) decreased while 33%, 33%, and 40% of them increased towards oocyte maturation respectively (Figs. 5, 6B). Regarding PCs, only 10–16% of the different lipid categories had a higher signal in females with mature oocytes, while 47–70% decreased their signal with the highest negative trend among PUFA PCs with 4–6 unsaturations (Figs. 5, 6C; Supplementary Table S2). On the contrary, a larger percentage of SFA (42%) and MFA (65%) TGs had an increasing signal in females during oocyte maturation (vs 25% and 17% with decreasing signal respectively), while most of the PUFA TGs (56%) decreased (Figs. 5, 6D). Only 22% of the PUFA TGs increased their signal during oocyte maturation (Figs. 5, 6D) most of these were with 2 and fewer than 3 unsaturations (Fig. 6D; Supplementary Table S2). Regarding other lipids, PEs (their methylated counterparts PMeE and PDMeE), LPE, PGs, and LPGs followed the same trend: their signal decreased in reproductive sponges (Supplementary Table S2; Supplementary Fig. S1). Finally, ether glycerophospholipids with PUFAs, such as etherPCs, etherPEs, etherPMeEs, etherPDMeEs also behaved similarly (Supplementary Table S2, Supplementary Fig. S1). Sphingolipids did not present a clear change with oocyte maturation (Figs. 5, 6E), but their signal rather decreased. Finally, oxylipins presented a general decreasing trend along oocyte maturation (Figs. 5, 6F). Remarkably, PGE_2_ and PGF_2,_ increased their signal with oocyte maturation (Fig. 6F).

### Gene expression patterns related to oogenesis and lipid metabolism

The total amount of sequenced raw reads was 189,570,217, while after filtering¸ 150,639,459 reads remained and were used for the de novo transcriptome assembly (Supplementary Table S3A). Our reference transcriptome had 574,591 transcripts, an N50 of 1240 and GC content of 45% (Supplementary Table S3B). The completeness of our assembly, based on BUSCO for metazoan cassettes, was 78.2% (for complete genes) and the overall alignment rate of raw reads to the reference transcriptome was 96.5% (Supplementary Table S3B). Roughly, 20.5% of the transcripts (117,682) were annotated against the Swiss-Prot database for metazoans (Supplementary Table S3B).

In the differential gene expression (DGE) analysis we obtained 165 upregulated genes in female individuals when compared to NR. Among those, 22.4% were annotated to a metazoan gene with known function (Supplementary file SF2; Supplementary Table S4; Supplementary Table S5A). The GO enrichment analysis revealed categories related to oogenesis and embryogenesis such as: “DNA methylation involved in gamete generation”, “female germline”, “ovarian follicle development”, “chorion” “instar larval development”, and “embryonic organ morphogenesis” (Supplementary Table S6A). Interestingly, the gene *low-density lipoprotein receptor* (*ldlr*), was overexpressed (Supplementary Table S5A).

**Figure 7:**
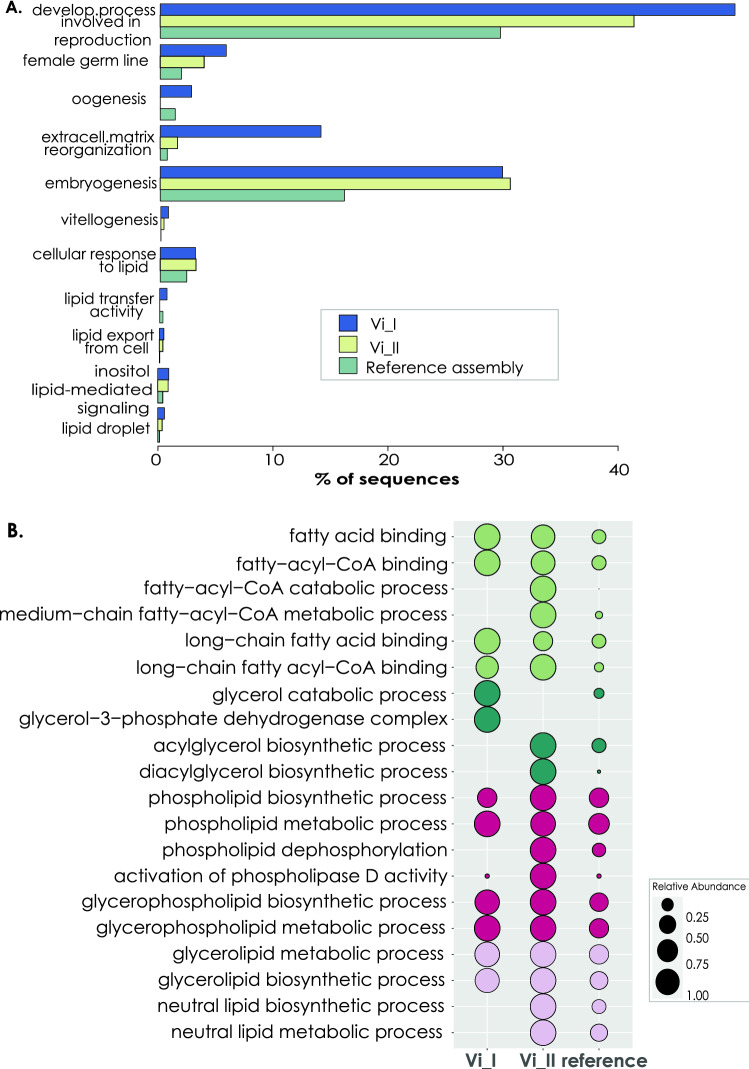
GO enriched categories in female specimens. (**A**) Barplot depicting the GO enriched categories related to oogenesis and female reproduction, based on the upregulated genes in females compared to nonreproductive individuals and the reference transcriptome. (**B**) Bubbleplot presenting GO enriched categories related to lipid metabolism derived from the upregulated genes in females compared to nonreproductive individuals. Each colour represents a different group of categories according to their function and the size of the circle in each case is relative to the expression level of this category. GO enrichment analysis was conducted selecting a *p* value ≤ 0.05.

When conducting the DGE analysis considering the females at different developmental stages, we obtained 3490 upregulated genes for Vi_I stage (vs NR) and 4774 for Vi_II (vs NR) (Supplementary file SF2; Supplementary Table S4), with 67.3% and 50.8% of those genes annotated respectively to metazoan genes (Supplementary Table S4; Supplementary Table S5B–C). In general, female individuals (either Vi_I or Vi_II), showed overexpressed genes that were associated to GO enriched categories related to oogenesis and female reproduction: “developmental process involved in reproduction”, “female germ-line”, “oogenesis”, “extracellular matrix reorganization”, “embryogenesis”, “vitellogenesis”, and categories related to response to lipid, lipid transfer and formation of lipid droplet (Fig. 7A; Supplementary Table S6B–C). In this line and focusing on lipid metabolism, there were several GO categories enriched either in Vi_I, Vi_II or both, when compared to the reference (the total annotation of the transcriptome assembly) that were related to fatty acid metabolism, glycerol and diacylglycerol metabolism, phospholipid/glycerophospholipid synthesis and metabolism and finally to TG biosynthesis and metabolism (Fig. 7B; Supplementary Table S6B–C).

**Figure 8:**
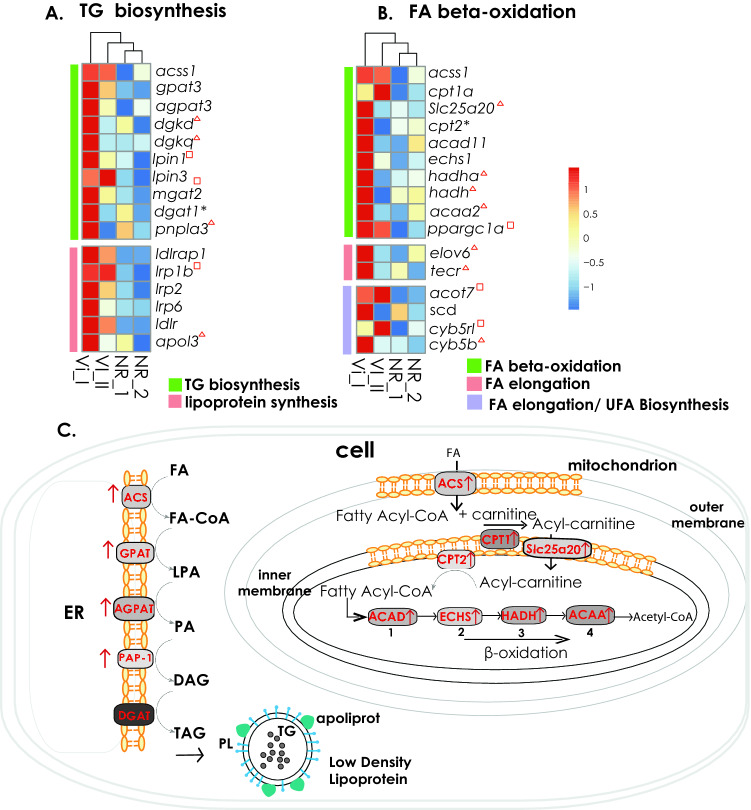
Gene expression patterns of lipid metabolism in *P. ventilabrum*. (**A**) Heatmap of genes regulating the triacylglyceride (TG) biosynthetic pathway and lipoprotein formation. (**B**) Heatmap of genes regulating the fatty acid (FA) beta-oxidation, FA elongation and synthesis of unsaturated fatty acids (UFA). Relative expression level increases from blue to red. DE genes were overexpressed in females (either vitellogenic I, Vi_I, or vitellogenic II, Vi_II) compared to nonreproductive specimens. The red triangle represents genes that were overexpressed only in Vi_I female while the red square represents genes that were overexpressed only in Vi_II female. The asterisks show genes that were not overexpressed; however, they were found in the expression matrix and showed a higher expression tendency in females. (**C**) Pathways of triacylglyceride biosynthesis in endoplasmic reticulum (ER), lipoprotein synthesis, fatty acid (FA) beta-oxidation in mitochondria, and the molecular regulators that activate the FA beta-oxidation. The red arrows indicate the overexpression of those genes in female individuals, either or Vi_I or Vi_II or both stages.

Our transcriptomic data revealed genes related to biosynthesis and metabolism of several of lipid categories identified by lipidomics (Supplementary Table S7) (see paragraph 2). KEGG pathways related to phospholipid/glycerophospholid and TG metabolism, fatty acid elongation but also fatty acid degradation and beta-oxidation were activated in female specimens (either Vi_I, Vi_II, or both) when compared to NR (Supplementary Table S8; Supplementary Fig. S2). In particular, almost all the genes participating in the TG biosynthetic pathway (*acetyl-coenzyme A synthetase*, *acss1*; *glycerol phosphate acyltransferase*, *gpat*; *acyl-glycerol-phosphate acyltransferase*, *agpat;* and *phosphatidate phosphatase, lpin3* coding for PAP-1) were overexpressed in females when compared to NR (Fig. 8A, C; Supplementary Table S5; Supplementary Table S7). Furthermore, genes related to fatty acid elongation and biosynthesis of unsaturated fatty acids, such as the *elongation of very long chain fatty acids protein 6, elov6*; *long-chain-fatty-acid-CoA ligase 5*, *acsl5*; *long-chain-fatty-acid-CoA ligase*, *acsbg2*; and the *very-long-chain enoyl-CoA reductase, tecr,* were also overexpressed in females (Fig. 8B–C; Supplementary Table S5A–B; Supplementary Table S7). Finally, all genes regulating all four steps of FFA beta-oxidation in mitochondria were also overexpressed in females vs NR: 1. dehydrogenation: *acyl-CoA dehydrogenase,* 2. hydration: *enoyl-CoA hydratase,* 3. oxidation: *hydroxyacyl*-*coenzyme A dehydrogenase,* 4. thiolysis: *etoacyl-CoA thiolase* (Fig. 8B–C; Supplementary Table S5B–C; Supplementary Table S7).

Several of the overexpressed genes in females are also implicated in arachidonic acid and linoleic acid metabolism (e.g. *calcium-independent phospholipase A2*, *pla2g6*, *group XV phospholipase A2*, *pla2g15, phospholipase abhd3, abhd3, 1-acylglycerol-3-phosphate O-acyltransferase*, *pnpla3)* (Supplementary Table S5; Supplementary Table S7), while interestingly, the gene *prostaglandin G/H synthase 2 (ptgs2*) and *peroxisomal acyl-coenzyme A oxidase 1* (*acox1*) related to alpha-linoleic acid metabolic processes were overexpressed in NR versus Vi_I or Vi_II individuals (Supplementary Table S5E–F, Supplementary Table S8). Finally, both upregulated and downregulated genes in female specimens are involved in KEGG pathways related to sphingolipid metabolism. For instance, upregulated genes were those coding for acid ceramidase (*asah1*), galactocerebrosidase (*galc*), serine palmitoyltransferase 2 (*sptlc2*), and ceramide synthase 1 (*cers1*) (Supplementary Table S5C–D), while downregulated genes included those coding for neutral ceramidase (*asah2*), putative sphingomyelin phosphodiesterase (*asm-3*), sphingosine-1-phosphate lyase (*sglA*), and sphingosine-1-phosphate lyase 1 (*sgpl1*) (Supplementary Table S5C–D).

## Discussion

In our study we found that the studied specimens of *Phakellia* *ventilabrum* were gonochoric and most possibly oviparous, reproducing in spring and end of summer/autumn in Western Norway and Western Sweden respectively, like most bubarids (see Busch et al.^107^ for other examples). Our comparative lipidomic and transcriptomic analysis shows that during oogenesis and at the stage of vitellogenesis, the signal of most TGs increases in concomitance with their de novo lipogenesis. In parallel, the signal of other lipid categories decreases and their beta-oxidation occurs. Our results therefore suggest that TGs might be the main component of the energy stock in the female gametes while other lipids undergo degradation to generate energy for the adult during this energetically high consuming process.

In both locations and sampling times, individuals with mature, vitellogenic oocytes were found among the collected specimens (Table 1), indicating either that this species has two annual reproductive cycles (one from February to May and another one from August to October) or that this species has different reproductive cycles in the different locations, triggered by local abiotic factors. In deep-sea habitats, where the rest of abiotic conditions are constant along the year, it has been stated that the seasonal primary production and the subsequent nutrients reaching the seafloor, influence the reproductive cycle of organisms^108,109^. Furthermore, previous studies have shown that the seasonal reproductive cycles of deep-sea sponges from the orders Polymastiida and Tetractinellida and the deep-sea coral *Desmophyllum pertusum* (Linnaeus, 1758) in boreo-arctic North-Atlantic were correlated to seasonal nutrient blooms^110,111^. In both sampling locations of our study, there is a spring planktonic bloom during March–April, and a smaller one during August-September^112,113^. So, even though it is difficult to narrow down the exact timing of the reproductive period of *P*. *ventilabrum,* with few samples from an extremely restricted period of time (and not collected on a monthly basis to properly monitor its reproductive cycle), we expect that its reproductive cycle coincides with that of other demosponge species, like *Geodia* spp. present at the same locations, and whose reproduction was triggered by abiotic conditions in these areas^83,111^. Spetland and collaborators^111^ found two reproductive cycles for populations of *Geodia barretti* Bowerbank, 1858 in the Kosterfjord (Western Sweden), with spawning seasons estimated to be in late spring (May/June) and October.

*Phakellia ventilabrum* is an oviparous species with potentially lecithotrophic larvae (as other members of closely related orders) or even direct development, so all the nutrients are accumulated in the egg during vitellogenesis. Here we observed that the oocyte maturation phase in specimens of *P*. *ventilabrum*, during which yolk accumulation occurs, was synchronized with the predictable increase of energy stocks in the surroundings, as noted previously for other deep-sea organisms^114,115^. The yolk within the oocytes of *P. ventilabrum* was heterogeneous, composed of proteins and lipids. We do not have any information on reproductive strategies of other sponge species from the same genus, with the exception of a study on *Phakellia hirondellei* Topsent, 1980^107^. The authors briefly reported only protein platelets within the oocyte of *P*. *hirondellei*. The closest phylogenetically sponge species being studied more extensively is the Mediterranean *Raspaciona aculeata* (Johnston, 1842) of the order Axinellida^116^ with heterogeneous yolk, mainly of protein origin described in the oocytes*.* Protein synthesis has proven costlier energetically in cold waters than in temperate environments for adult isopods^117^, but the cost for protein synthesis was very low during the early developmental stages of the Antarctic sea urchin *Sterechinus neumayeri* (Meissner, 1900)^118^. There is no study calculating the metabolic cost of protein and lipid synthesis in sponges from cold habitats. However, lipid was the only type of yolk present in the Antarctic sponge *Mycale* (*Oxymycale*) *acerata* Kirkpatrick, 1907 and was considered an adaptation to cold environments since its Caribbean counterpart *M*. (*Mycale*) *laevis* (Carter, 1882) had mainly heterogeneous yolk^55^. Similarly, *Geodia* spp. from the boreo-arctic deep-sea contained much more lipid yolk^110^ than *Geodia cydonium* (Linnaeus, 1767) from shallow temperate waters^119^. From the above we conclude that the yolk origin in *P*. *ventilabrum* is governed both by phylogenetic constrains and adaptation to its boreal water environment, revealing higher amounts of lipid yolk than its more temperate counterparts*.*

Reproduction is energetically a very costly process, and many changes in lipid metabolism have been observed in marine invertebrates during this period, with some lipids increasing and others decreasing^20,120^. Indeed, some lipid categories constitute the nutrients for the future embryo (lipid droplets formed during phase of vitellogenesis) and they increase in female individuals suggesting their de novo synthesis and other lipids provide energy resources for the adult, so they are catabolized during gamete formation (oocyte growth, differentiation, nutrient formation and nutrient transport). Our lipidomic analysis revealed that sponges remodel their lipidome during oogenesis following a tendency to (i) increase TGs with SFAs and MFAs, (ii) decrease glycerophospholipids, (iii) decrease PUFAs with a high number of unsaturations and (iv) increase the beta-oxidation of FFAs.

Almost half of the detected SFA and MFA TGs showed a tendency to increase their signal from NR towards the females with mature oocytes (Figs. 5, 6D) suggesting that this type of lipid is the main component of yolk in this species. TGs store much more energy (10 times higher/gram) than any other type of lipids and carbohydrates^120^, and are the most compacted form of energy storage^121^. In marine organisms, TGs, together with wax esters (WE) and sterols (ST), are the main forms of energy storage^10,33,122^ and are very common in deep-sea fauna, including sponges^2^. While cnidaria prefer WE/ST as storage lipids, deep-sea sponges use both TGs and WE/ST to store their energy^2^. Interestingly, TGs together with phospholipids (PLs) also provide the most common source of energy storage in the gametes and reproductive organs of terrestrial^123–126^ and many marine invertebrates^18,127–130^. This supports our hypothesis for the role of TGs in yolk storage in the oocytes of *P*. *ventilabrum*.

SFA and MFA TGs, e.g. TG(49:0), TG(58:0), TG(52:0), TG(54:0), TG(50:1), TG(48:1), TG(52:1), which had the highest tendency to increase their signal along oogenesis, are associated with de novo lipogenesis^131^. In parallel, we observed a decrease in several free fatty acids that constitute these TGs, such as FA(16:0) or FA(18:0) (Fig. 6A), which can be explained by a more extensive incorporation of these fatty acids into TGs. Most of the genes regulating the enzymes of the TG biosynthetic pathway (most of which also participate in PL biosynthetic pathway)^132,133^ were overexpressed in females with vitellogenic oocytes (either Vi_I or Vi_II or both), confirming the occurrence of de novo lipogenesis in female individuals, likely engaged in yolk formation (Fig. 8; Supplementary Table S7; Supplementary Table S8). Only the gene expressing the penultimate enzyme in the TG biosynthesis, *diacylglycerol acyltransferase (dgat),* was not significantly overexpressed, but still more expressed in females with Vi_I oocytes (Fig. 8A, C). GO enriched categories and KEGG pathways related to the long chain FAs and elongation of FAs were enriched; other genes from these pathways were also upregulated in females (Fig. 8B; Supplementary Table S5, Supplementary Table S8; Supplementary Fig. S2). These are possibly related to TG biosynthesis as TGs contain particularly long-chain FAs^134^. In general, SFA and MFA FAs can generate a higher equivalent of ATP^135^ and this suggests that the enrichment of TGs with less unsaturated fatty acids is associated with a higher energy accumulation during reproduction.

In our analysis, even though the majority of PLs were decreasing their signal as oogenesis progressed, ~ 20% of the PLs exhibited an increased signal (Figs. 5, 6, Supplementary Table S2), indicating that they may have a role in vitellogenesis but in a more selective way than TGs. Our data suggest that different lipids participate in the formation of different fractions (homogeneous lipid platelets/ heterogeneous lipid-protein platelets) of the lipid yolk in sponges, but further more targeted analyses are needed to confirm this observation. Histochemical analysis in vitellogenic oocytes of the lecithotrophic lizard *Zootoca vivipara*, Lichtenstein, 1823, (*Lacerta vivipara* in the publication), showed that phospholipids were deposited in yolk in association with protein in heterogenous droplets, whereas triglycerides were found in separate deposits^136^. Another role of PLs in yolk formation is the construction of lipoproteins for TG transport. TGs have a hydrophobic nature and they are transported extracellularly or intracellularly in animals in the form of lipoproteins^137^. Even though there are no studies discussing the presence and role of lipoproteins in sponges, it is known that lipoproteins have appeared early in evolution^137^ and have been detected in ovaries of marine invertebrates, playing a role in lipid transport^16,138^. In our transcriptomic data, GO enriched categories (“very-low-density lipoprotein particle assembly” and “lipoprotein particle binding”) and overexpressed genes related to lipoprotein formation (*low-density lipoprotein receptor-related protein, lrp; low density lipoprotein receptor adapter protein 1, ldlrap1; low-density lipoprotein receptor, ldlr;* and *apolipoprotein L3, apol3*) in females, compared to NR (see Results section) (Fig. 8A, C; Supplementary Table S5; Supplementary Table S6; Supplementary Table S7), strengthen the hypothesis that lipid transport, including TG transport, in sponges occurs also with the help of lipoproteins as in the sea anemone *Nematostella vectensis* Stephenson, 1935^138^. Furthermore, vitellogenin receptors, which are lipoproteins related to yolk formation in the oocytes of egg-laying species^139^, have been identified in *P. ventilabrum* and other demosponges^98,140^. In our study, genes for both vitellogenin and its receptor were three times more expressed in the Vi_I female than NR, although they were not differentially expressed.

The largest variation of PUFAs was observed in samples between the different locations/months (Fig. 4), indicating that these lipids are mostly related to nutrients in the environment. However, during oogenesis it was observed that PUFAs with ≥ 3 unsaturations, including PUFA TGs, decreased (Fig. 6; Supplementary Table S2). These lipids are used/oxidized to provide fast energy to the adult because they are more unstable than the SFAs or MFAs. Sponge specimens that do not reproduce, potentially, store them for other future physiological functions and/or consume them at a slower pace than the reproductive specimens, and that could be why we observed a higher signal in specimens without oocytes in their mesohyl (Fig. 6D). The mechanism of PUFA oxidation in sponges is not known but we could not find any upregulated genes related to mammal PUFA oxidation^141^. In addition the decrease of FA(22:6), (normally produced after PUFA oxidation in mammals^142^) and of their oxylipins (HDoHEs), do not suggest either an increase of the PUFAs oxidation, at least in a similar way to what happens in mammals. It might be the case that sponges follow a different mechanism of PUFA oxidation, and further studies are needed to investigate this.

While the PUFA oxidation route in sponges might be novel, it is also possible that PUFAs in sponges are oxidized via the regular FA oxidation route in mitochondria. Indeed, we found a higher activity of genes related to FA beta-oxidation in females compared to NR (Fig. 8B–C, Supplementary Fig. S2), including activation and transportation of FA to the mitochondria by *acs*; *carnitine:palmitoyltransferase*, *cpt*; and mitochondrial *carnitine/acylcarnitine carrier protein*, *Slc25a20*^143^ and their beta-oxidation to produce acyl-CoA, the main component entering the citric cycle. This overexpression indicates that there was an increase in FA beta-oxidation due to high energetic requirements during oogenesis. Indeed, most of the carbon accumulated in sponges (90%) is spent in generating energy for physiological processes such as growth, pumping and reproduction (Koopmans, 2009), and lipids are a large source used for oxidation in sponges as they consist of more than 50% of the dry weight of the particulate organic matter that sponges feed on^144^. Therefore, we cannot discard alternative routes for lipid oxidation to provide energy in sponges. The extra enzymes required for the oxidation of unsaturated FA, either for odd or even number of carbons (*Delta(3,5)-Delta(2,4)-dienoyl-CoA isomerase*, *ech1*; and *2,4-dienoyl-CoA reductase*, *decr1*) were detected in our analysis, however, without overexpression in females. This suggests either that these FA are absorbed for production of other lipids, or that, again, there is a different mechanism for oxidising these lipids in sponges.

Lastly, oxylipins seem to have a negative correlation with the female reproduction in our study, and only the prostaglandins PGE_2_ and PGF_2a_ increased in their signal as oocytes developed (Fig. 6F). Prostaglandins (PGs) have a signalling role in many physiological processes, including reproduction. PGs and PGE_2_ specifically, play a role in oocyte maturation and egg formation in mammals^145–147^. They also play a role in egg production and/or laying of the eggs in marine invertebrates such as molluscs^148^ and in crickets^149^. In corals, PGs are involved in defence mechanisms while in sponges they have been found to play a role in cell motility, differentiation, proliferation and cell aggregation^150,151^. So far, no specific studies have tested the specific role of PGs in sponge reproduction. However, due to the fact that PGs are well conserved along the animal kingdom^152^and as PGE_2_ increased with the size of oocytes and their maturity in our data, one can speculate that there is a potential role of PGE_2_ in the reproduction of *P. ventilabrum*. However, the determination of cause-effect relationships in the reproduction of *P. ventilabrum* was beyond the scope of this study and further studies would be needed to study deeper this hypothesis.

This is the first study in which the reproductive strategy of the sponge *Phakellia ventilabrum,* a keystone species of the vulnerable deep-sea sponge grounds of North Atlantic is investigated. It is also the first application of semi-targeted lipidomics to a sponge, which allowed us to detect more sponge lipids than any other previous work. Interestingly, we detected lipids that are absent or in low amounts in mammals, such as phytosphingolipids and glycerophospholipids of methylethanolamine or dimethylethanolamine. Further studies with a focus on targeted gametic cells would be necessary to understand in depth the processes of lipid synthesis, lipid transport, and oxidation during oogenesis in Porifera. In addition, the characterisation of the specific structure of lipids and the tracing with isotope labelling would enhance our comprehension on the role of nutrient uptake from the surrounding to the pathways of FA incorporation for synthesis of other lipids, to the yolk formation during oogenesis in sponges, and their route of oxidation.

## Conclusions

Our study has important ecological and evolutionary implications. Information on the reproductive activity and strategy of *P*. *ventilabrum* enhances our understanding of ecosystem functions of sponge grounds and can contribute to develop conservation strategies in these areas. Furthermore, our findings illustrate the outstanding diversity of the sponge “chemical dark matter” often difficult to comprehend, and that new metabolomic methods are just starting to reveal. The insights of lipid metabolism combining lipidomics and transcriptomics during oogenesis in sponges, given for first time in this study, further provide essential information in order to understand the origin and molecular basis of vitellogenesis in Metazoa. Finally, our results also give hints on the fitness of the propagules and how imbalances in lipid resources in the environment could affect the fitness of the species.

## Supplementary Information

Supplementary Legends.

Supplementary Table S1.

Supplementary Information 1.

Supplementary Table S2.

Supplementary Figure S1.

Supplementary Table S3.

Supplementary Information 2.

Supplementary Table S4.

Supplementary Table S5.

Supplementary Table S6.

Supplementary Table S7.

Supplementary Table S8.

Supplementary Figure S2.

Supplementary Table S9.

## Data Availability

The raw reads generated for the transcriptomic analysis of this study were deposited at Sequence Read Archive (SRA) with BioProject ID: PRJNA64180 and submission ID: SUB7667177.
