# Supplementary material for: Oogenesis and lipid metabolism in the deep-sea sponge Phakellia ventilabrum (Linnaeus, 1767)
Source: Sci Rep. 2022 Apr 15;12:6317. doi: 10.1038/s41598-022-10058-6 (PMC9012834; doi:10.1038/s41598-022-10058-6)
Supplement: Supplementary file 1 — Supplementary Legends. [file 41598_2022_10058_MOESM1_ESM.docx]

**Supplementary material**

**Table S1**. **Lipid identification and lipid signal ((au)/mg of dry sponge) of the different lipid categories detected** **in the studied individuals of *Phakellia ventilabrum***, including different developmental stages and different locations/months studied. Table indicates **A.** the Lipid identification and the signal of: **B.** Fatty acids (FA), **C.** phosphatidylcholines (PC) and lysophosphatidylcholines (LPC), **D.** phosphatidylethanolamines (PE) and lysophosphatidylethanolamines (LPE), **E.** phospatidylglycerols (PG) and lysophosphatidylglycerols (LPG), **F.** triacylglycerides (TG), **G.** sphingolipids and glycosphingolipids, **H.** oxylipins.

**SF1.** **Comparison of signal intensity [au/mg] of lipids among the different locations/Months.** PCA for each lipid category and boxplots of all the different lipids within each group are depicted, indicating the significant variation (in orange) of the lipids after comparison with the statistical t-test. The lipid category of fatty acids (FA), phosphatidylcholines (PC), and lysophosphatidylcholines (LPC), phosphatidylethanolamines (PE) and lysophosphatidylethanolamines (LPE), phosphatidylglycerols (PG) and lysophosphatidylglycerols (LPG), triacylglycerides (TG), sphingolipids and glycosphingolipids were analysed

**Table S2.** **Coefficient Regression Analysis**. Lipids from all different lipid categories with a negative or positive coefficient in relationship with the increasing area of sponge tissue occupied by oocytes

**Figure S1.** Relationship between lipids and the area of sponge tissue occupied by oocytes for several lipid categories. The x-axis represents the number of unsaturations of the lipids and the y-axis the coefficient that relates the signal of the lipid with the surface of oocytes. Lipids above the dashed line were upregulated with oocytes, lipids below this line were downregulated with oocytes. To distinguish major from minor species in the same family, the area of the points is proportional to the signal of the lipid in the control group. The number next to the points indicates the number of carbons of the lipid

**Table S3. Transcriptomic data. A.** Number of raw and trimmed reads after filtering for the four sequenced individuals of *P. ventilabrum*. **B.** Statistical data of the transcriptome and the blast hits.

**SF2. Transcriptomic data.** Heatmap and volcano plots depicting the differential expressed genes among the different comparisons, Vi_I vs NR and Vi_II vs NR. Abbreviations: NR, nonreproductive; Vi_I*,* vitellogenic I, Vi_II, vitellogenic II.

**Table S4. Differentially expressed genes (DE):** N of differentially expressed genes between the pairwise comparisons: genes that are up- or downregulated in female with vitellogenic stage I (Vi_I) or vitellogenic stage II (Vi_II) oocytes *vs* nonreproductive specimens. N of differentially expressed genes with blast ID: number of the up or downregulated genes which have a blast hit against Swiss-Prot database for Metazoa.

**Table S5. Differentially expressed genes (DE):** list of overexpressed genes in: **A.** female with vitellogenic stage I (Vi_I) oocytes compared to nonreproductive (NR) individuals. **B.** female with vitellogenic stage II (Vi_II) oocytes compared to nonreproductive (NR) individuals. **C.** NR individuals compared to female with Vi_I oocytes. **D.** NR individuals compared to female with Vi_II oocytes.

**Table S6. Gene Ontology (GO) enrichment analysis of the genes upregulated during oogenesis. A.** enriched GO categories in female individuals with vitellogenic stage oocytes (either vitellogenic stage I or vitellogenic stage II), when compared to nonreproductive individuals **B.** enriched GO categories in female individual with vitellogenic stage I (Vi_I) oocytes, when compared to nonreproductive individuals. **C.** enriched GO categories in female individual with vitellogenic stage II (Vi_II) oocytes, when compared with nonreproductive individuals. The GO categories included cellular component, biological process and molecular function. The analysis was performed with threshold p-value ≤0,05, using as reference the annotation file extracted from the assembly of each species.

**Table S7. DE genes.** List of differentially expressed genes related to lipid metabolism and discussed within the study.

**Table S8. Activated KEGG pathways.** Lists of activated KEGG pathways from overexpressed genes in female with Vi_I stage oocytes and female with Vi_II stage oocytes when compared to nonreproductive individuals, indicating the enzymes participating in these pathways, the regulated pathways and the genes that express these enzymes.

**Figure S2.** **Activated KEGG pathways related to lipid metabolism;** derived from the overexpressed genes in female individuals, either of Vi_I (Vitellogenic I), Vi_II (Vitellogenic II) or both females when compared to nonreproductive (NR) individuals.

**Table S9.** **A.** dried material from P. ventilabrum used for the lipid and oxylipin analyses. **B.** Gradient for lipid chromatographic separation.
