# Supplementary material for: Oogenesis and lipid metabolism in the deep-sea sponge Phakellia ventilabrum (Linnaeus, 1767)
Source: Sci Rep. 2022 Apr 15;12:6317. doi: 10.1038/s41598-022-10058-6 (PMC9012834; doi:10.1038/s41598-022-10058-6)

Fatty Acids

SFA_Fatty Acids

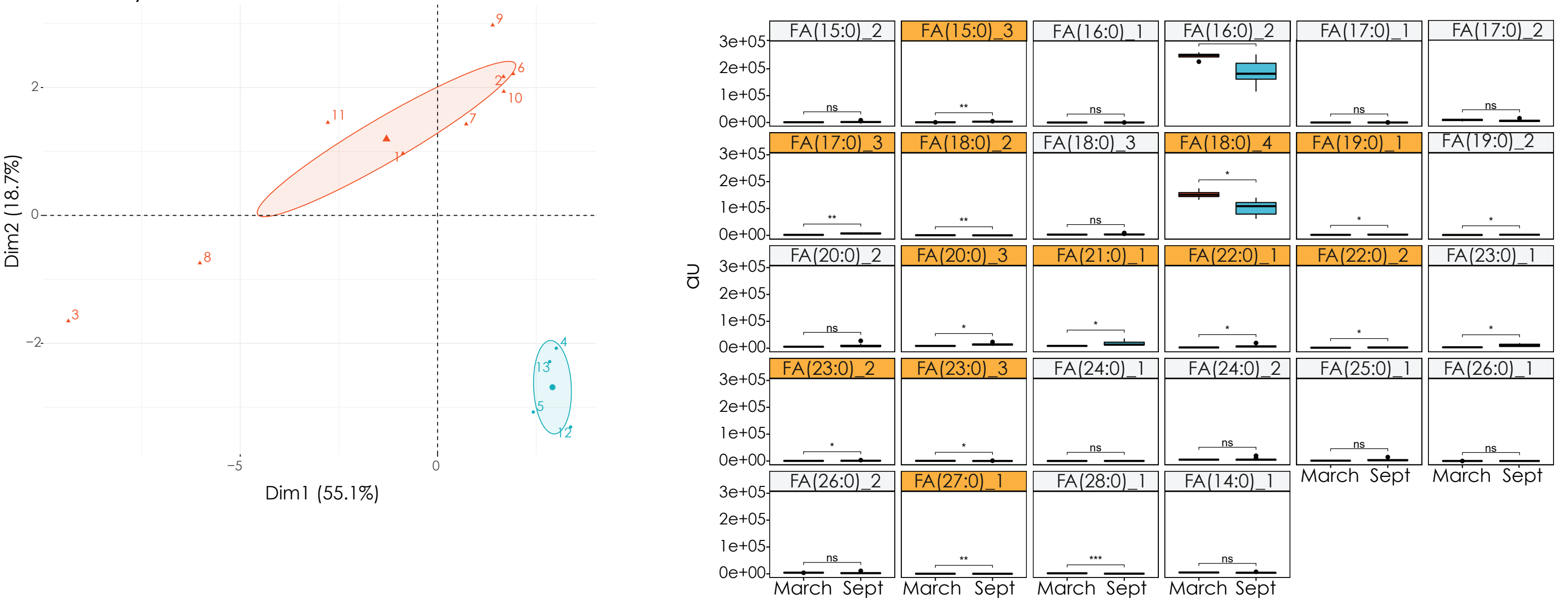

MFA_Fatty Acids

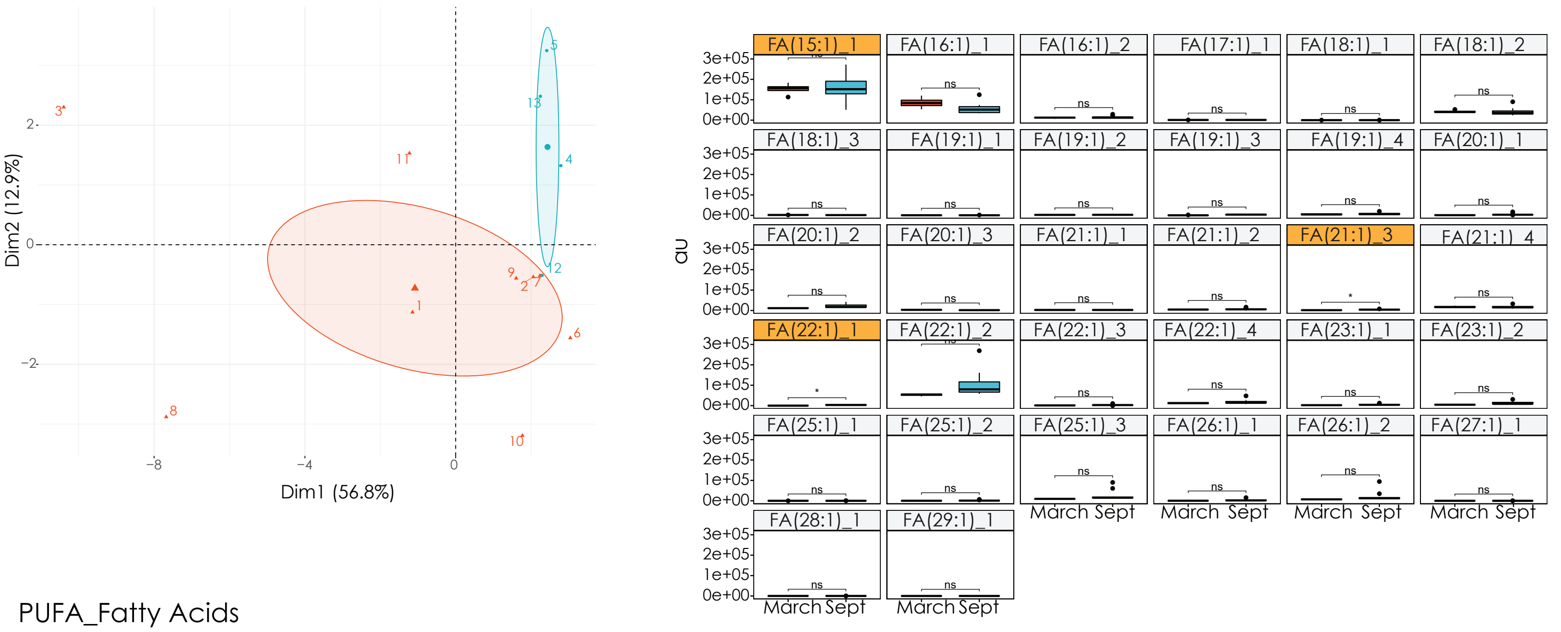

PUFA_Fatty Acids

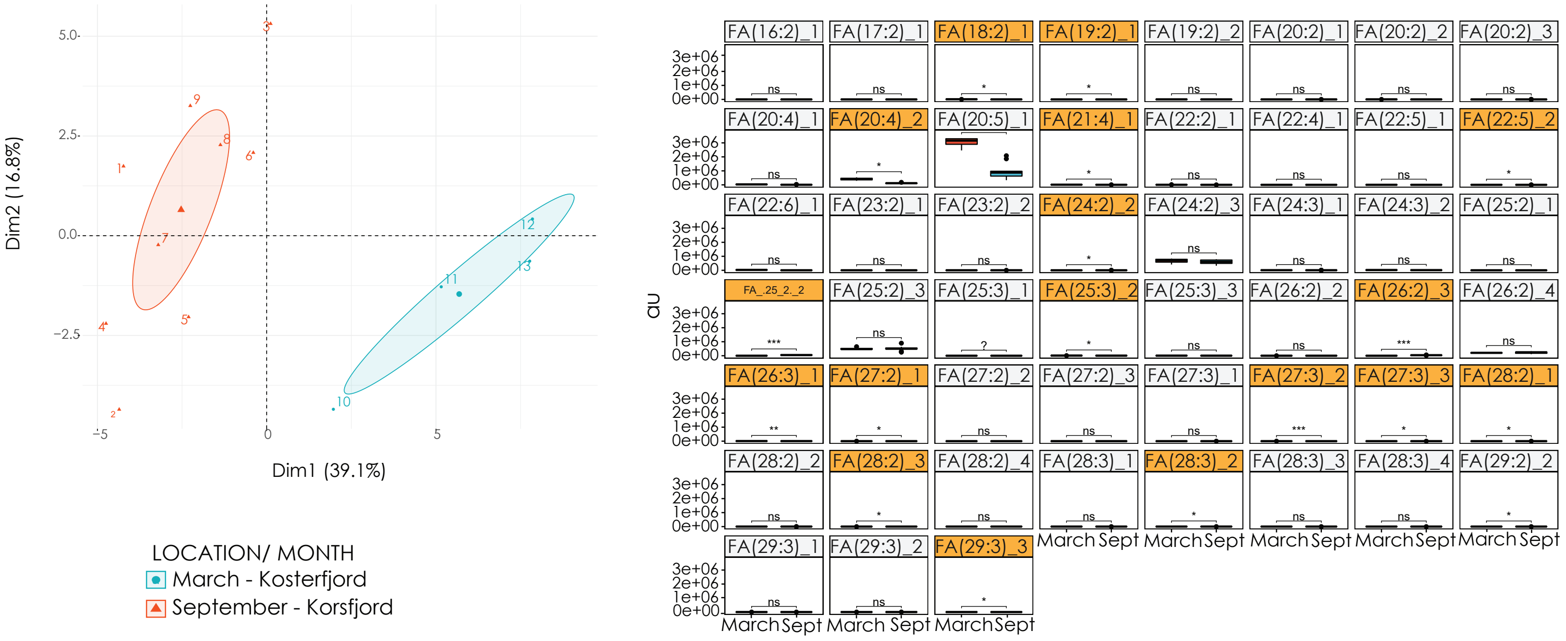

Phosphatidylcholines

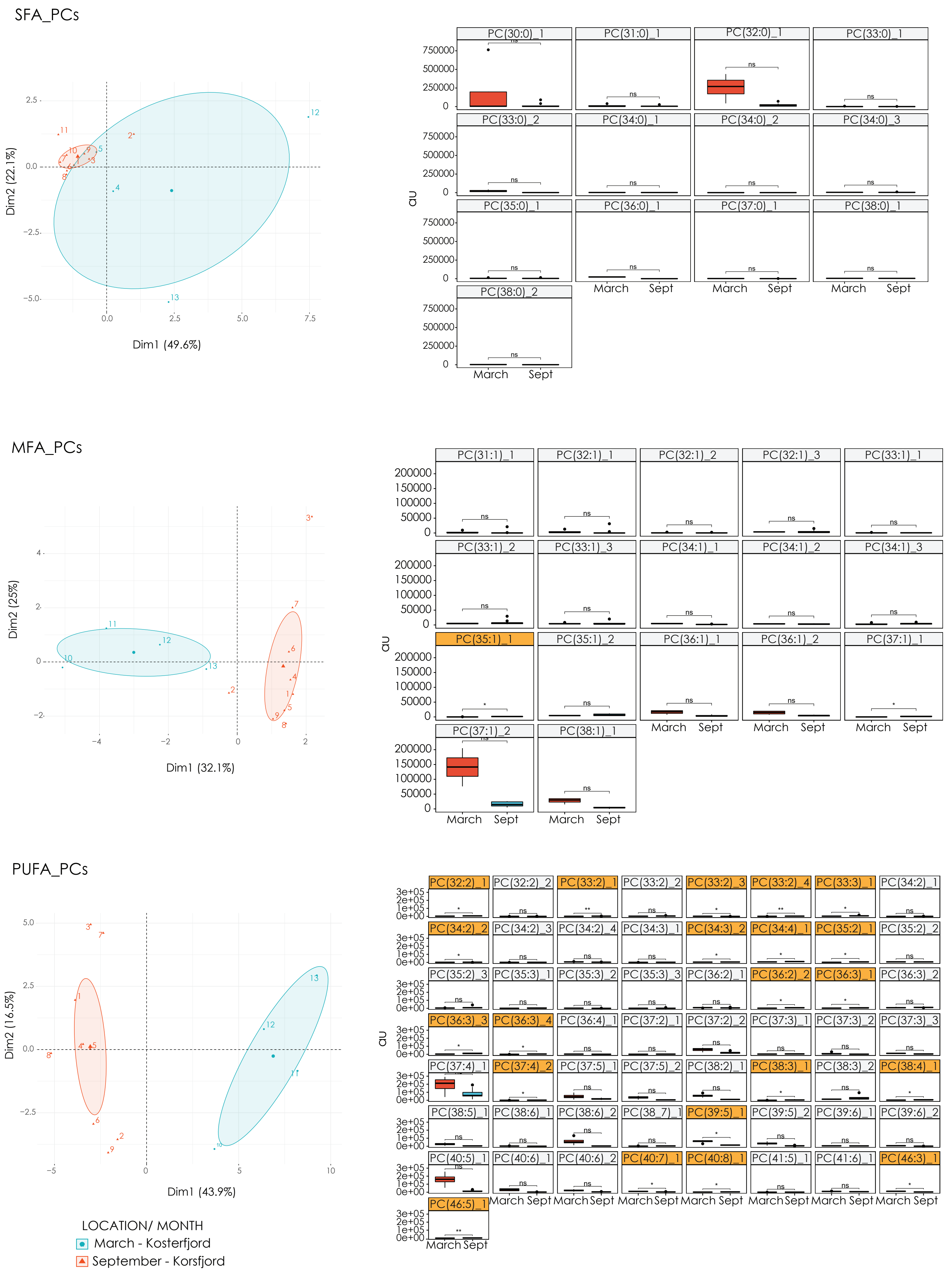

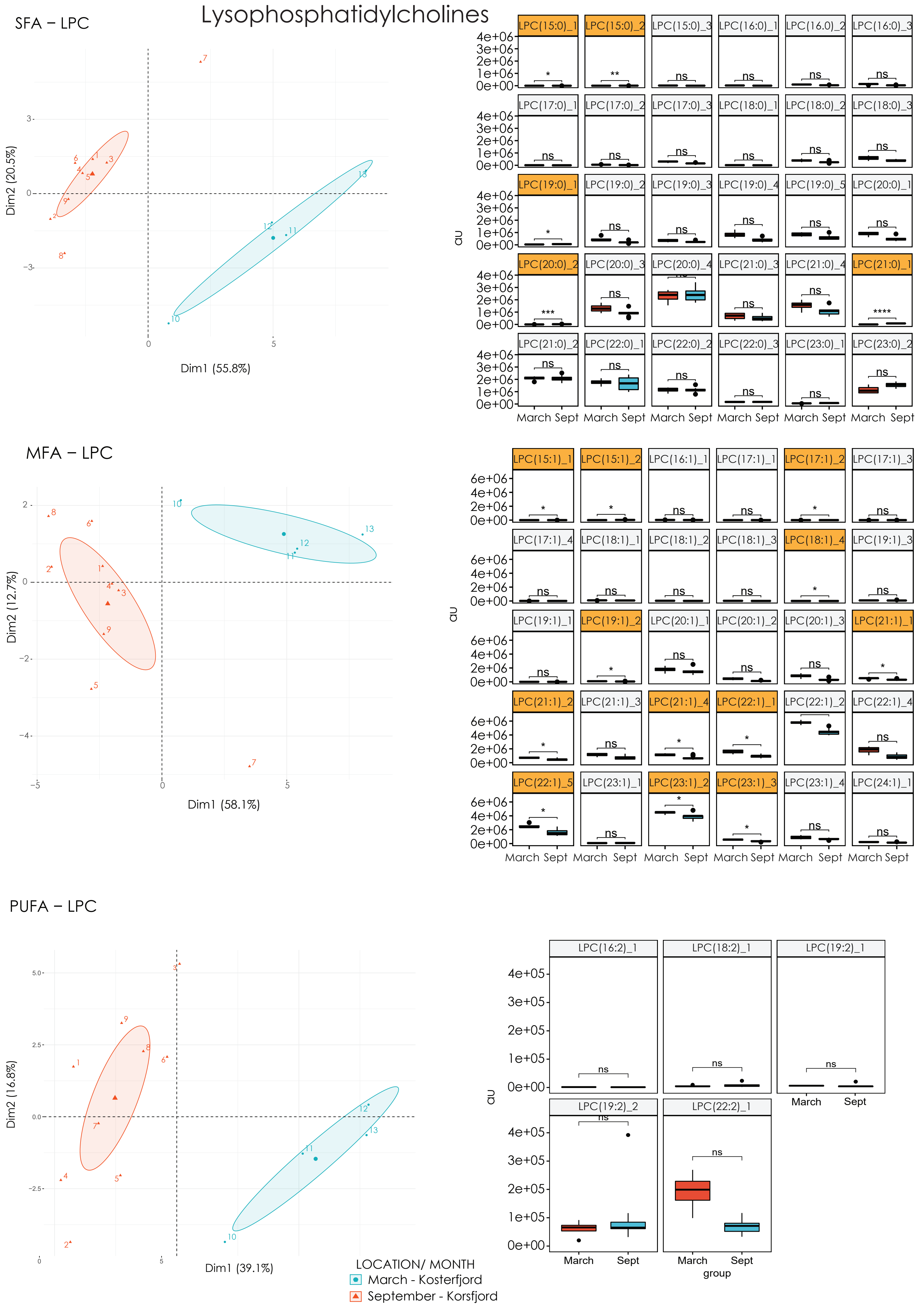

Lysophosphatidylcholines
SFA – LPC
Dim1 (55.8%)
Dim2 (20.5%)
LPC(15:0)_1 LPC(15:0)_2 LPC(15:0)_3 LPC(16:0)_1 LPC(16.0)_2 LPC(16:0)_3
LPC(17:0)_1 LPC(17:0)_2 LPC(17:0)_3 LPC(18:0)_1 LPC(18:0)_2 LPC(18:0)_3
LPC(19:0)_1 LPC(19:0)_2 LPC(19:0)_3 LPC(19:0)_4 LPC(19:0)_5 LPC(20:0)_1
LPC(20:0)_2 LPC(20:0)_3 LPC(20:0)_4 LPC(21:0)_3 LPC(21:0)_4 LPC(21:0)_1
LPC(21:0)_2 LPC(22:0)_1 LPC(22:0)_2 LPC(22:0)_3 LPC(23:0)_1 LPC(23:0)_2
MFA – LPC
Dim1 (58.1%)
Dim2 (12.7%)
LPC(15:1)_1 LPC(15:1)_2 LPC(16:1)_1 LPC(17:1)_1 LPC(17:1)_2 LPC(17:1)_3
LPC(17:1)_4 LPC(18:1)_1 LPC(18:1)_2 LPC(18:1)_3 LPC(18:1)_4 LPC(19:1)_3
LPC(19:1)_1 LPC(19:1)_2 LPC(20:1)_1 LPC(20:1)_2 LPC(20:1)_3 LPC(21:1)_1
LPC(21:1)_2 LPC(21:1)_3 LPC(21:1)_4 LPC(22:1)_1 LPC(22:1)_2 LPC(22:1)_4
LPC(22:1)_5 LPC(23:1)_1 LPC(23:1)_2 LPC(23:1)_3 LPC(23:1)_4 LPC(24:1)_1
PUFA – LPC
Dim1 (39.1%)
Dim2 (16.8%)
LPC(16:2)_1 LPC(18:2)_1 LPC(19:2)_1 LPC(19:2)_2 LPC(22:2)_1
au
March Sept
group
LOCATION/ MONTH
March - Kosterfjord
September - Korsfjord

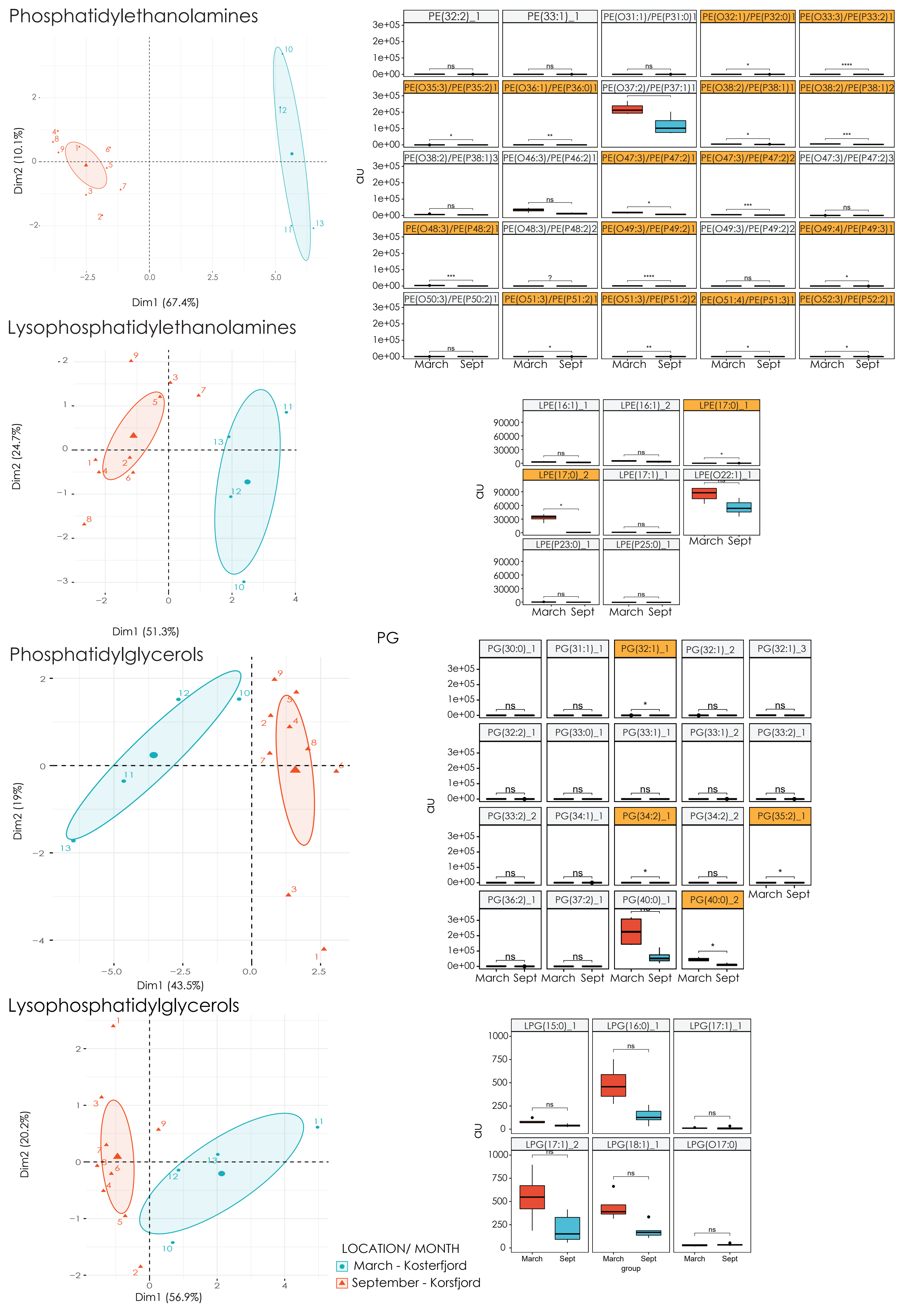
Phosphatidylethanolamines
Dim1 (67.4%)
Dim2 (10.1%)
PE(32:2)_1
PE(33:1)_1
PE(O31:1)/PE(P31:0)1
PE(O32:1)/PE(P32:0)1
PE(O33:3)/PE(P33:2)1
PE(O35:3)/PE(P35:2)1
PE(O36:1)/PE(P36:0)1
PE(O37:2)/PE(P37:1)1
PE(O38:2)/PE(P38:1)1
PE(O38:2)/PE(P38:1)2
PE(O38:2)/PE(P38:1)3
PE(O46:3)/PE(P46:2)1
PE(O47:3)/PE(P47:2)1
PE(O47:3)/PE(P47:2)2
PE(O47:3)/PE(P47:2)3
PE(O48:3)/PE(P48:2)1
PE(O48:3)/PE(P48:2)2
PE(O49:3)/PE(P49:2)1
PE(O49:3)/PE(P49:2)2
PE(O49:4)/PE(P49:3)1
PE(O50:3)/PE(P50:2)1
PE(O51:3)/PE(P51:2)1
PE(O51:3)/PE(P51:2)2
PE(O51:4)/PE(P51:3)1
PE(O52:3)/PE(P52:2)1
au
March Sept
Lysophosphatidylethanolamines
Dim1 (51.3%)
Dim2 (24.7%)
LPE(16:1)_1
LPE(16:1)_2
LPE(17:0)_1
LPE(17:0)_2
LPE(17:1)_1
LPE(O22:1)_1
LPE(P23:0)_1
LPE(P25:0)_1
PG
Phosphatidylglycerols
Dim1 (43.5%)
Dim2 (19%)
PG(30:0)_1
PG(31:1)_1
PG(32:1)_1
PG(32:1)_2
PG(32:1)_3
PG(32:2)_1
PG(33:0)_1
PG(33:1)_1
PG(33:1)_2
PG(33:2)_1
PG(33:2)_2
PG(34:1)_1
PG(34:2)_1
PG(34:2)_2
PG(35:2)_1
PG(36:2)_1
PG(37:2)_1
PG(40:0)_1
PG(40:0)_2
Lysophosphatidylglycerols
Dim1 (56.9%)
Dim2 (20.2%)
LPG(15:0)_1
LPG(16:0)_1
LPG(17:1)_1
LPG(17:1)_2
LPG(18:1)_1
LPG(O17:0)
group
LOCATION/ MONTH
March - Kosterfjord
September - Korsfjord

Triacylglycerides

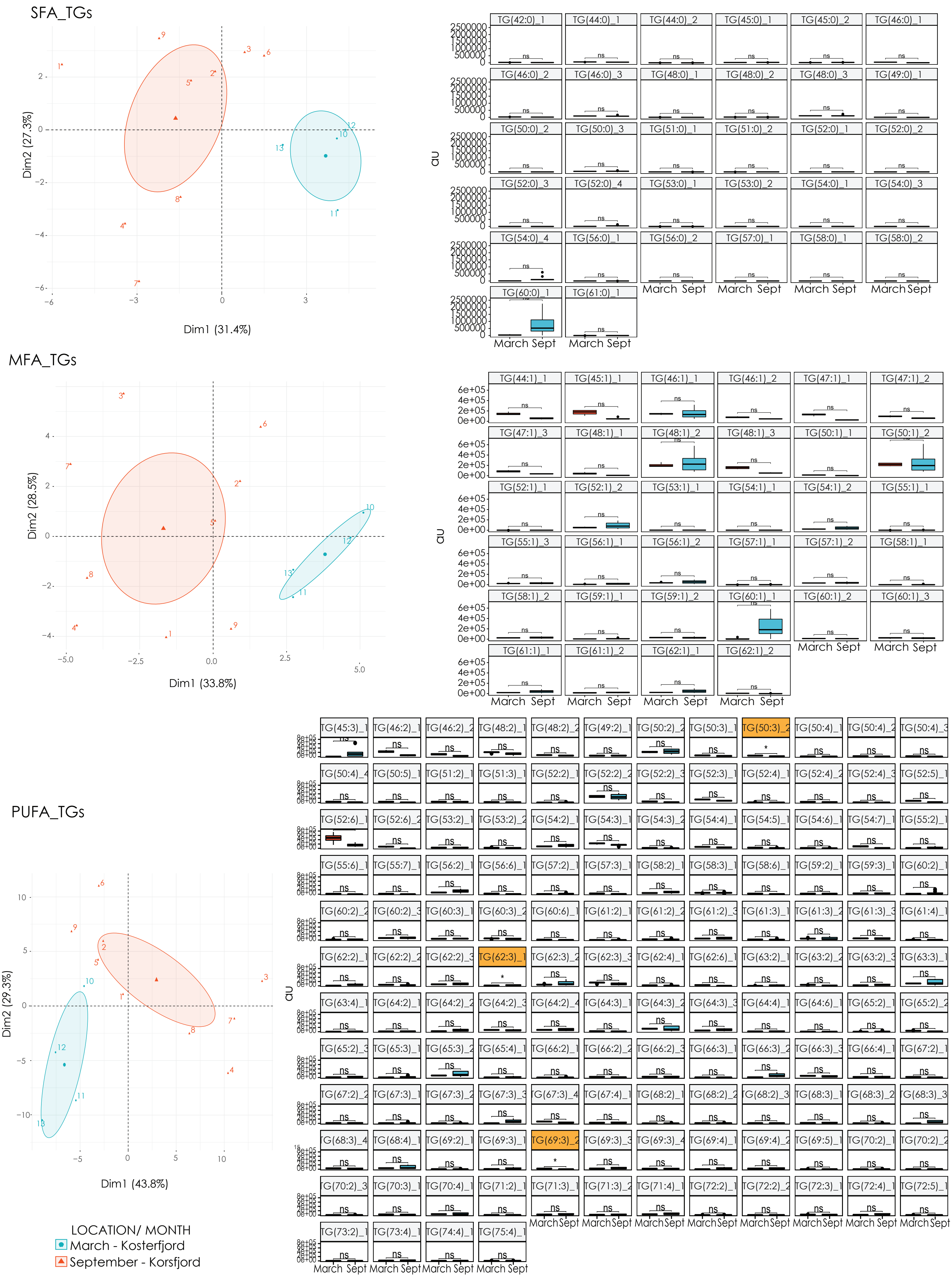

Sphingolipids-glycosphingolipids

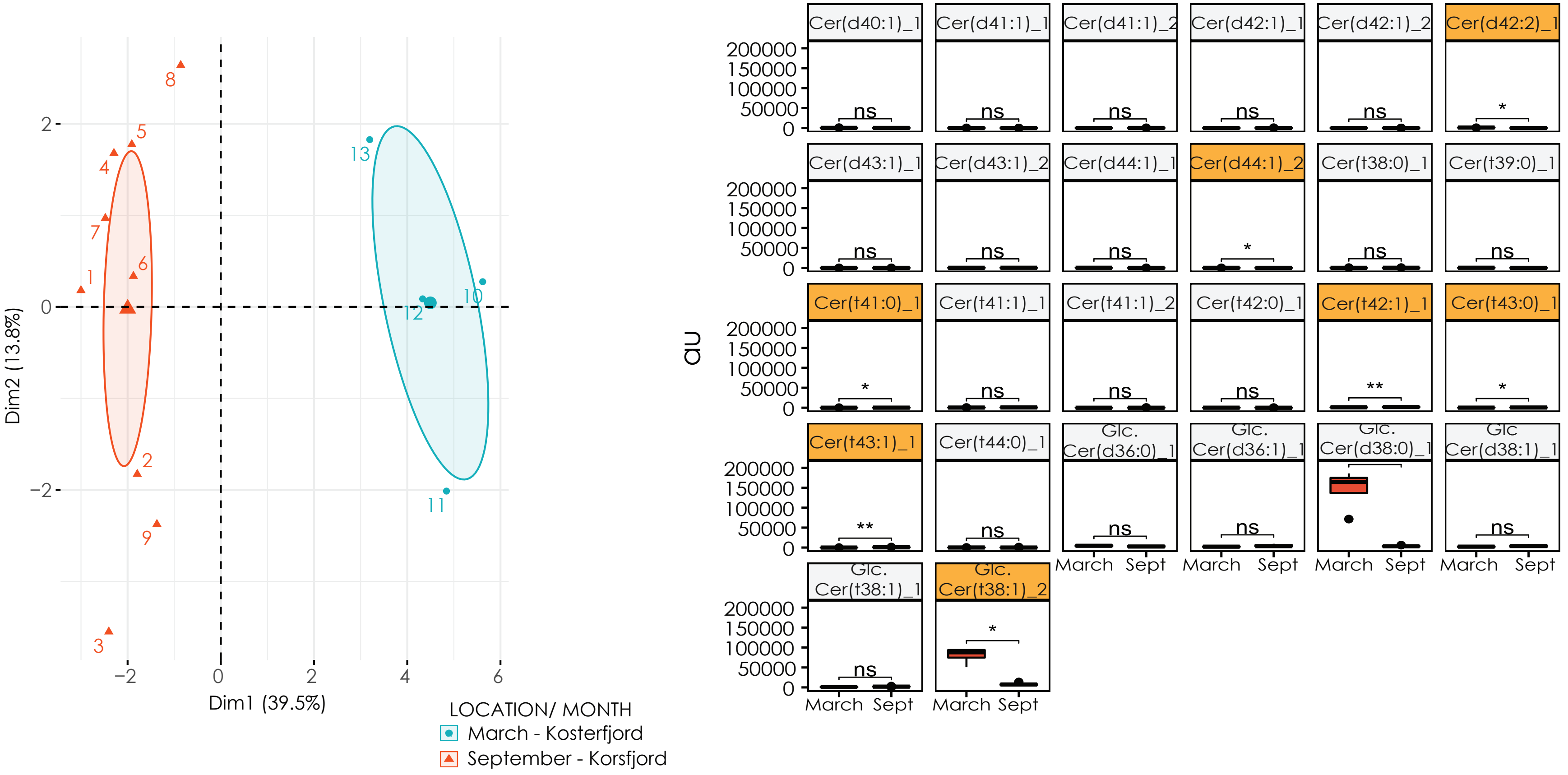

Supplement: Supplementary file 3 — Supplementary Information 1. [file 41598_2022_10058_MOESM3_ESM.pdf]
