# Supplementary material for: Oogenesis and lipid metabolism in the deep-sea sponge Phakellia ventilabrum (Linnaeus, 1767)
Source: Sci Rep. 2022 Apr 15;12:6317. doi: 10.1038/s41598-022-10058-6 (PMC9012834; doi:10.1038/s41598-022-10058-6)
Supplement: Supplementary file 5 — Supplementary Figure S1. [file 41598_2022_10058_MOESM5_ESM.pdf]

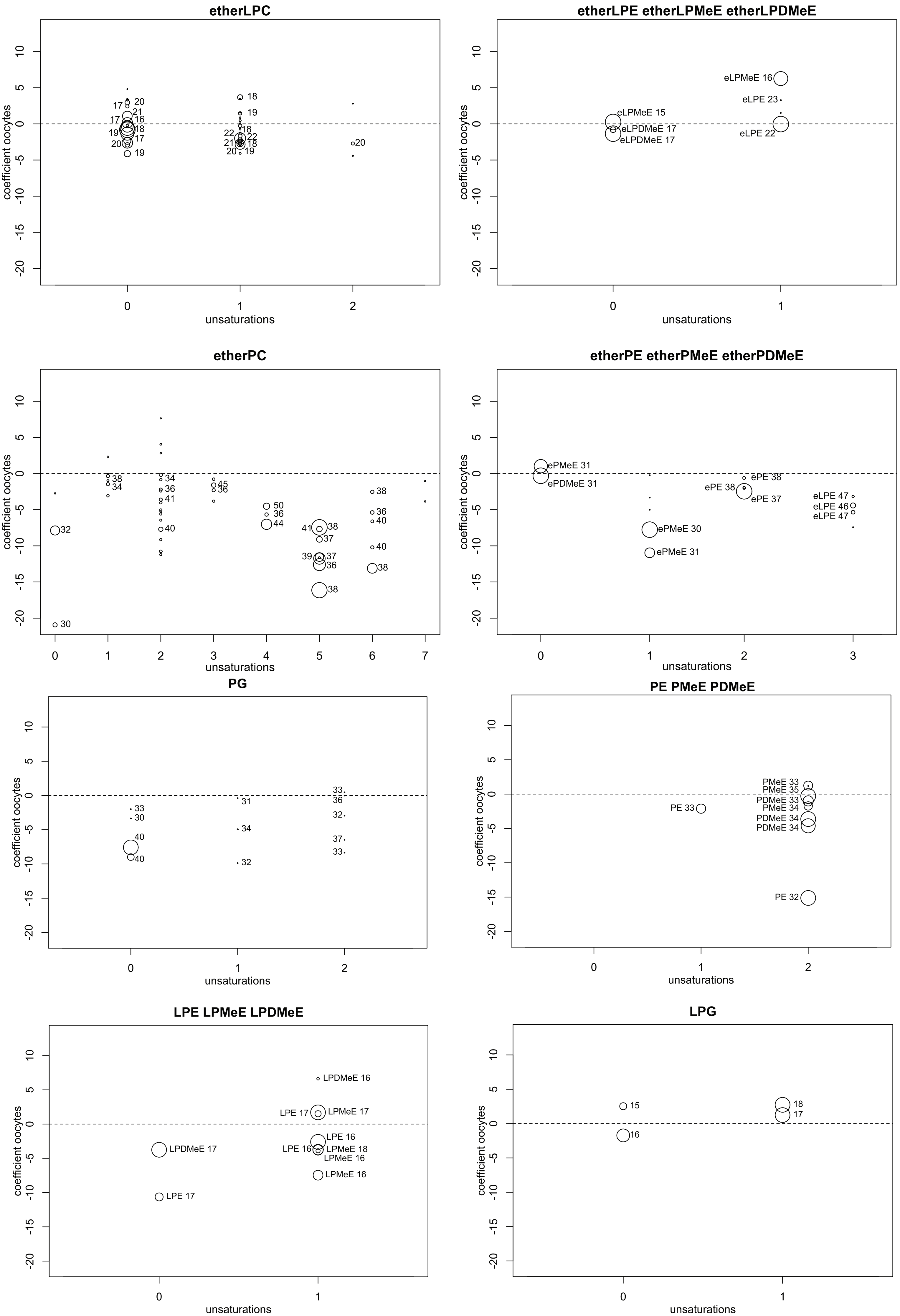
etherLPC
coefficient oocytes
unsaturations
17 20
21
17 16
18
19
17
20
19
18
19
22 18
21 22
18
20 19
20
etherLPE etherLPMeE etherLPDMeE
eLPMeE 16
eLPE 23
eLPMeE 15
eLPDMeE 17
eLPDMeE 17
eLPE 22
etherPC
38
34
34
36
41
40
45
36
50
36
44
41 38
37
39 37
36
38
38
36
40
40
38
32
30
etherPE etherPMeE etherPDMeE
ePMeE 31
ePDMeE 31
ePE 38
ePE 38
ePE 37
eLPE 47
eLPE 46
eLPE 47
ePMeE 30
ePMeE 31
PG
33
30
40
40
31
34
32
33
36
32
37
33
PE PMeE PDMeE
PMeE 33
PMeE 35
PDMeE 33
PMeE 34
PDMeE 34
PDMeE 34
PE 33
PE 32
LPE LPMeE LPDMeE
LPDMeE 16
LPE 17
LPMeE 17
LPE 16
LPE 16
LPMeE 18
LPMeE 16
LPMeE 16
LPDMeE 17
LPE 17
LPG
18
17
15
16
