# Supplementary material for: Oogenesis and lipid metabolism in the deep-sea sponge Phakellia ventilabrum (Linnaeus, 1767)
Source: Sci Rep. 2022 Apr 15;12:6317. doi: 10.1038/s41598-022-10058-6 (PMC9012834; doi:10.1038/s41598-022-10058-6)
Supplement: Supplementary file 7 — Supplementary Information 2. [file 41598_2022_10058_MOESM7_ESM.pdf]

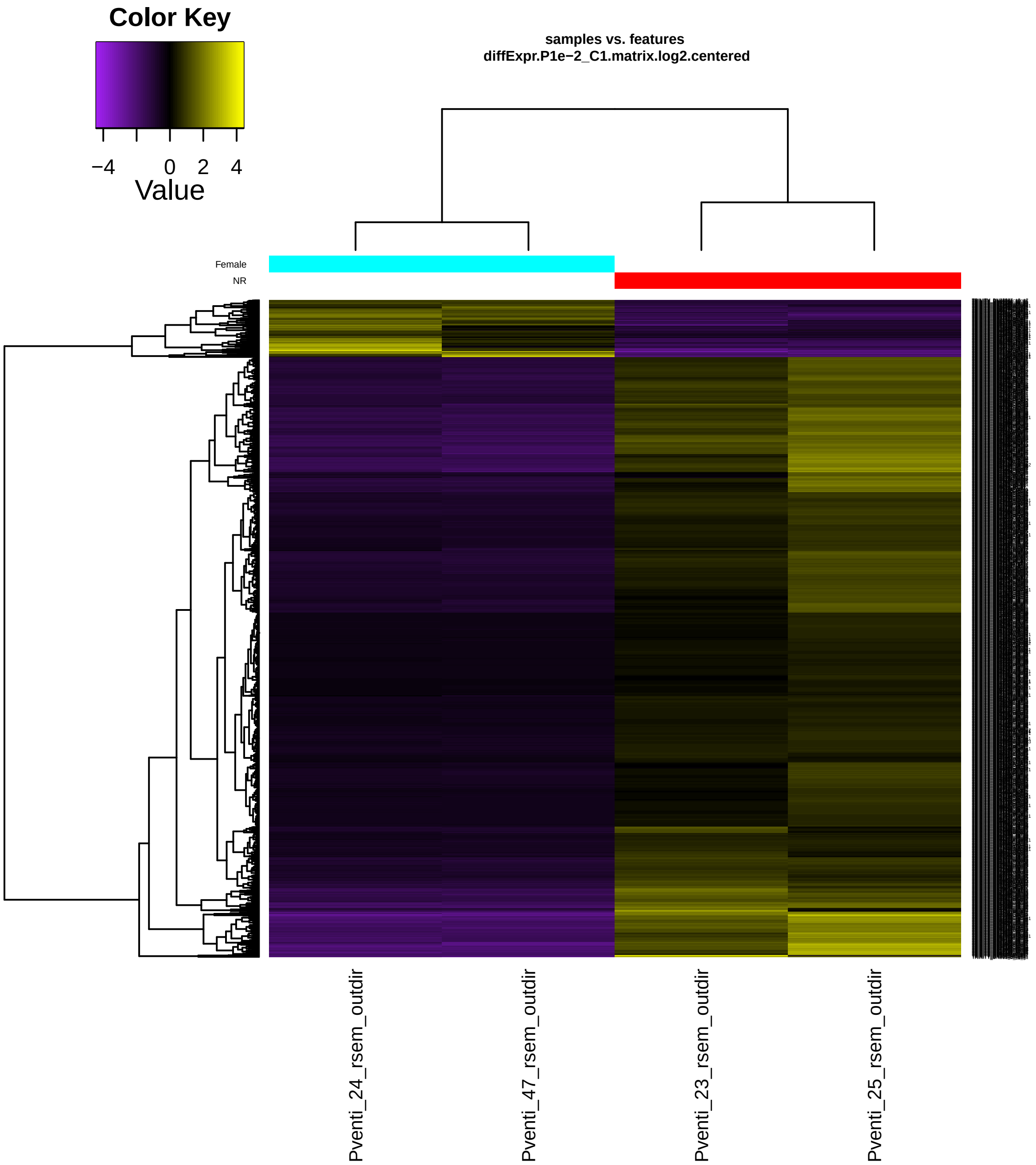
Color Key
−4
0
2
4
Value
samples vs. features
diffExpr.P1e−2_C1.matrix.log2.centered
Female
NR
Pventi_24_rsem_outdir
Pventi_47_rsem_outdir
Pventi_23_rsem_outdir
Pventi_25_rsem_outdir

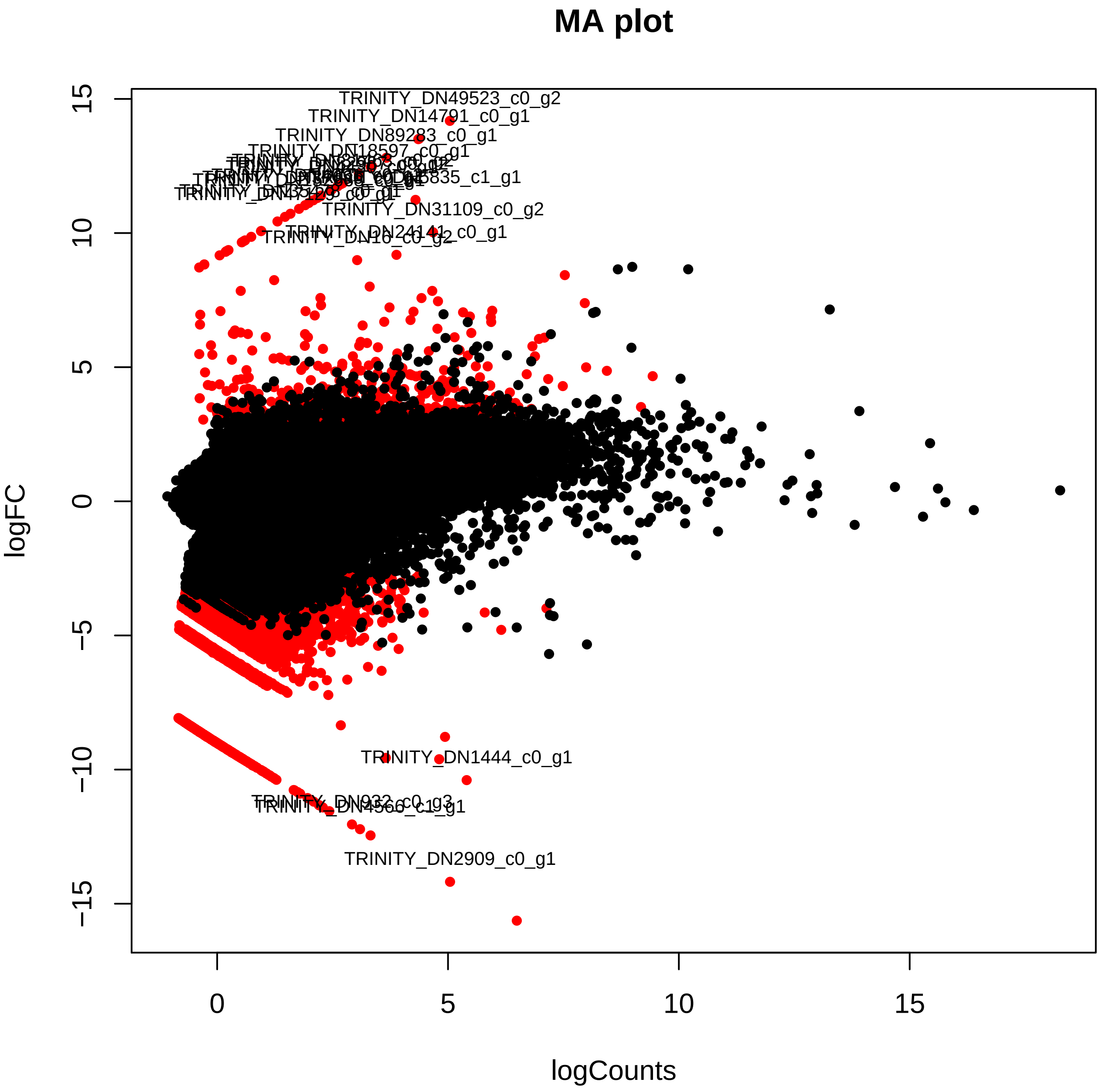
MA plot
TRINITY_DN49523_c0_g2
TRINITY_DN14791_c0_g1
TRINITY_DN89283_c0_g1
TRINITY_DN18597_c0_g1
TRINITY_DN31109_c0_g2
TRINITY_DN1444_c0_g1
TRINITY_DN2909_c0_g1
logFC
logCounts
15
10
5
0
−5
−10
−15
0
5
10
15

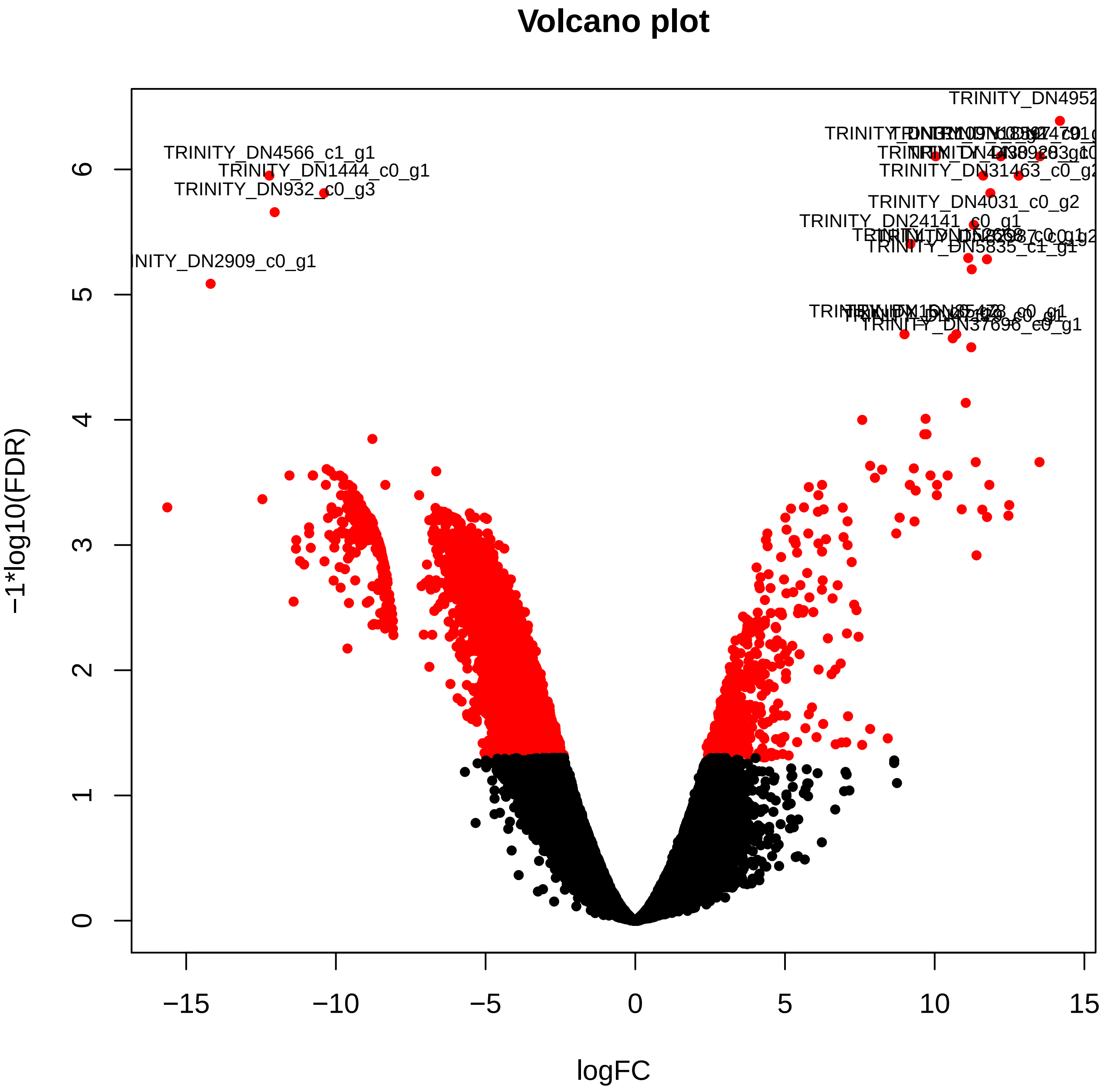
Volcano plot
TRINITY_DN4566_c1_g1
TRINITY_DN1444_c0_g1
TRINITY_DN932_c0_g3
NITY_DN2909_c0_g1
TRINITY_DN31463_c0_g2
TRINITY_DN4031_c0_g2
TRINITY_DN24141_c0_g1
TRINITY_DN5835_c1_g1
TRINITY_DN37696_c0_g1
−1*log10(FDR)
logFC
0
1
2
3
4
5
6
−15
−10
−5
0
5
10
15

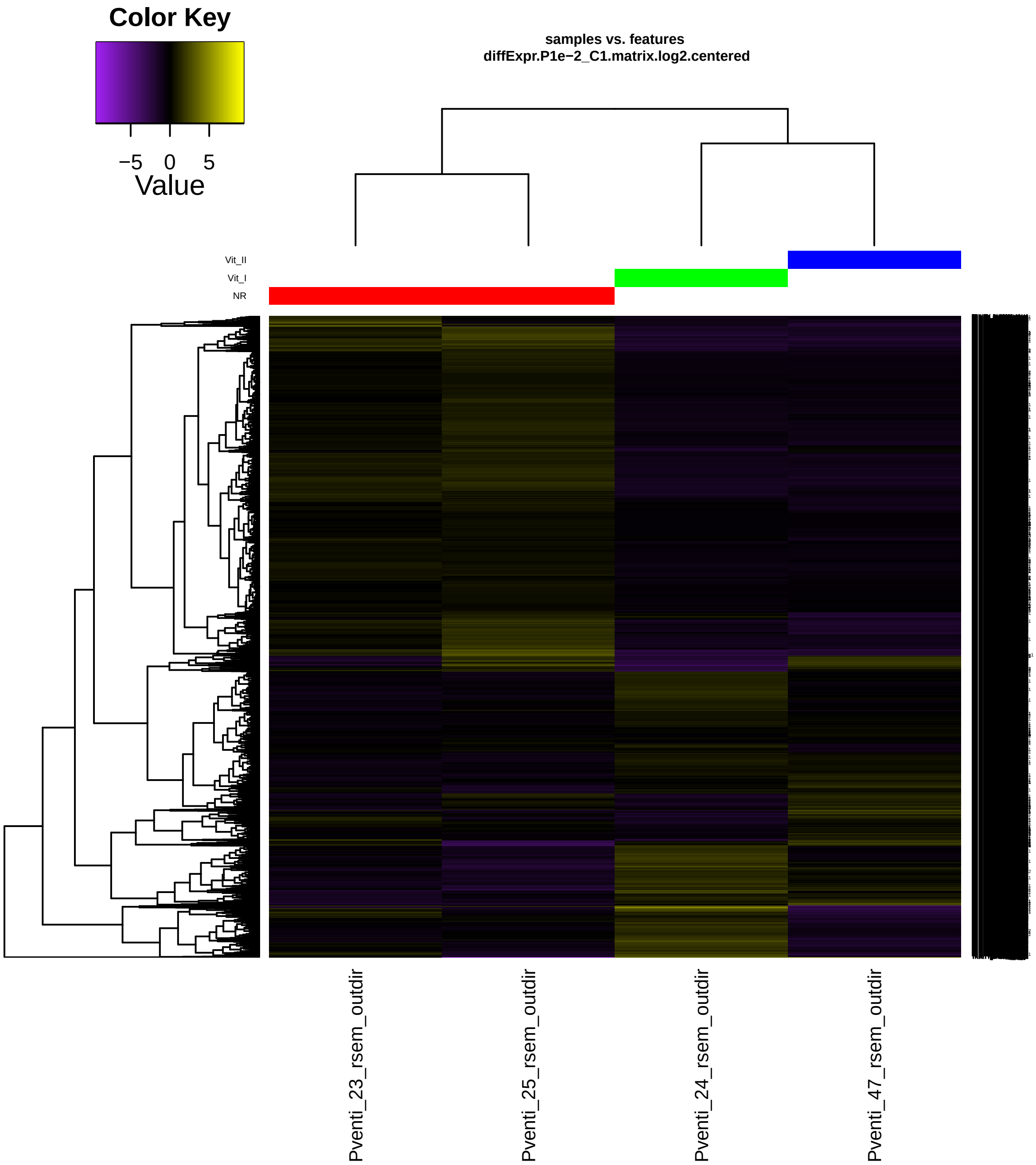
Color Key
−5 0 5
Value
samples vs. features
diffExpr.P1e−2_C1.matrix.log2.centered
Vit_II
Vit_I
NR
Pventi_23_rsem_outdir
Pventi_25_rsem_outdir
Pventi_24_rsem_outdir
Pventi_47_rsem_outdir

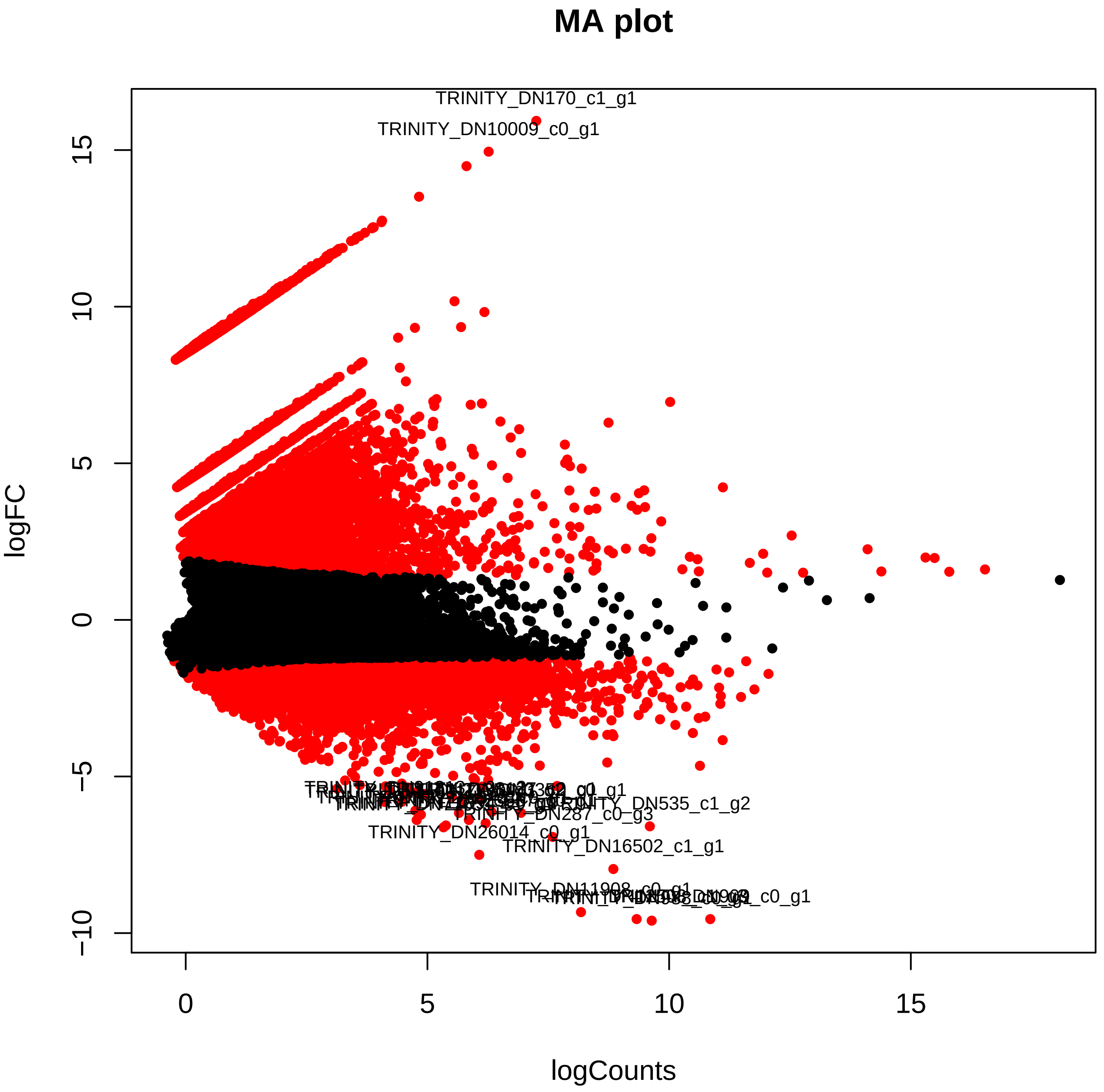
MA plot
logFC
logCounts
15
10
5
0
−5
−10
0
5
10
15
TRINITY_DN170_c1_g1
TRINITY_DN10009_c0_g1
TRINITY_DN535_c1_g2
TRINITY_DN287_c0_g3
TRINITY_DN26014_c0_g1
TRINITY_DN16502_c1_g1
TRINITY_DN11908_c0_g1

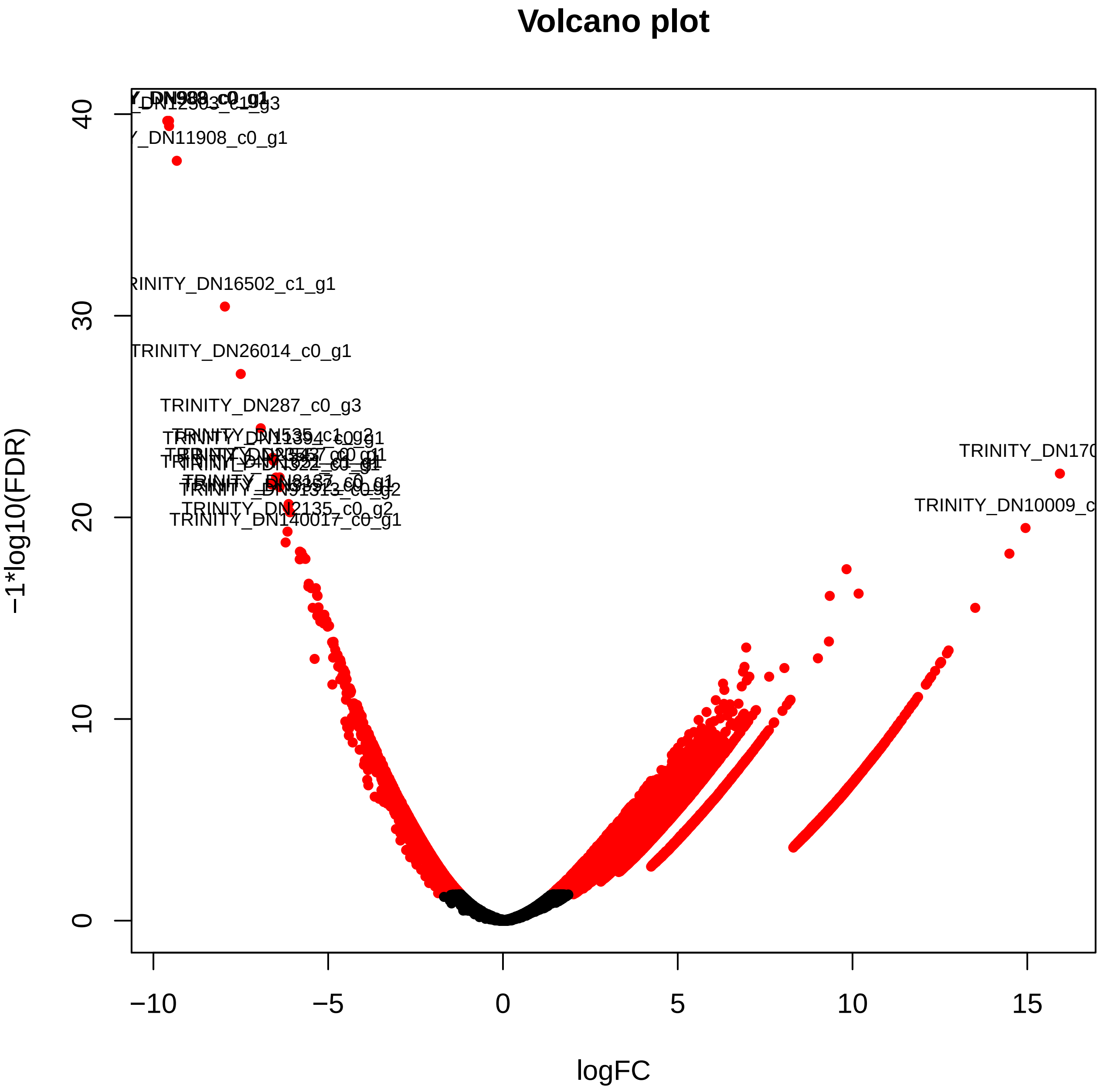

Volcano plot
−1*log10(FDR)
logFC
0
10
20
30
40
−10
−5
0
5
10
15
Y_DN11908_c0_g1
RINITY_DN16502_c1_g1
TRINITY_DN26014_c0_g1
TRINITY_DN287_c0_g3
TRINITY_DN2135_c0_g2
TRINITY_DN140017_c0_g1
TRINITY_DN170
TRINITY_DN10009_c

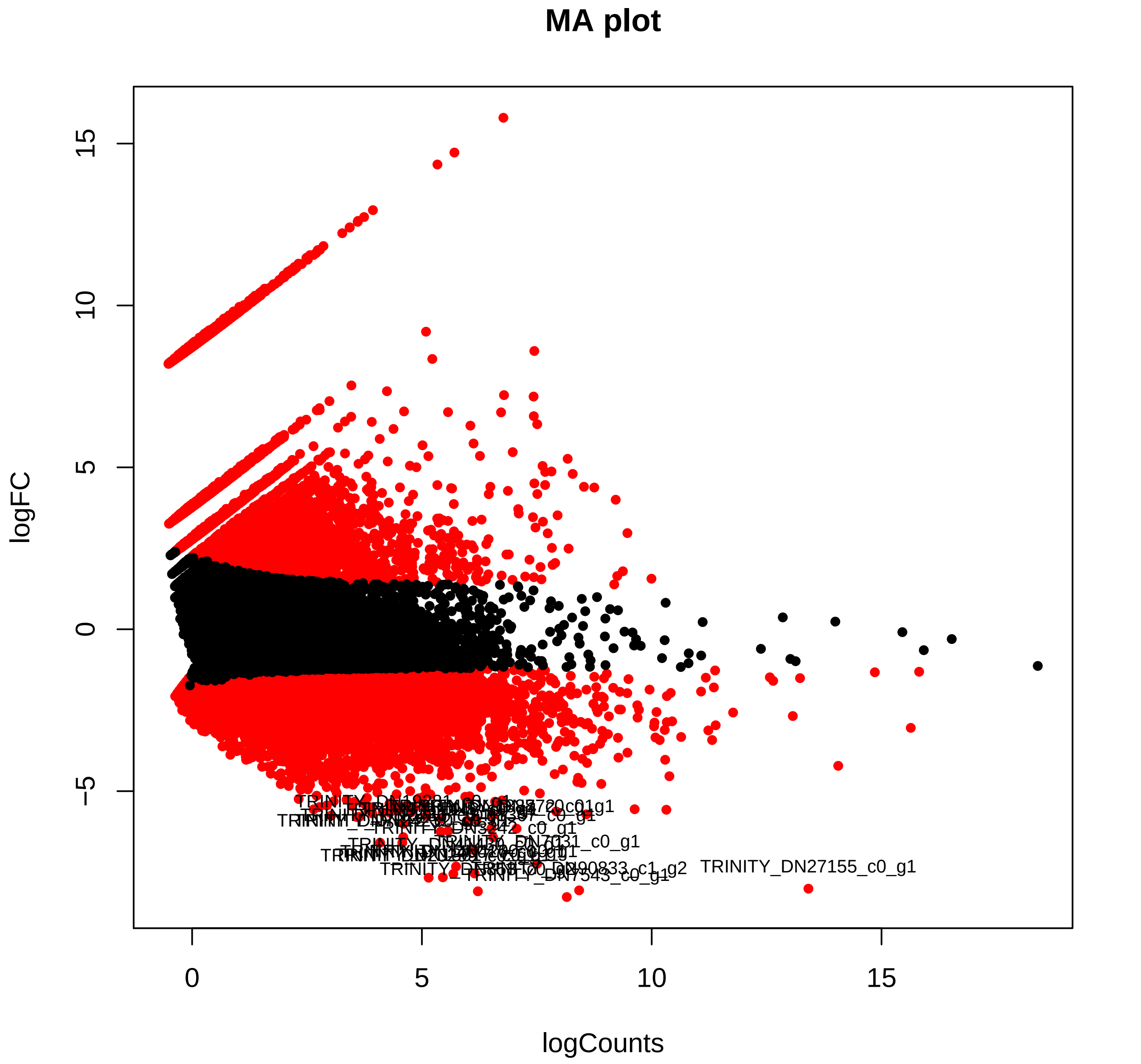
MA plot
logFC
logCounts
15
10
5
0
−5
0
5
10
15
TRINITY_DN27155_c0_g1

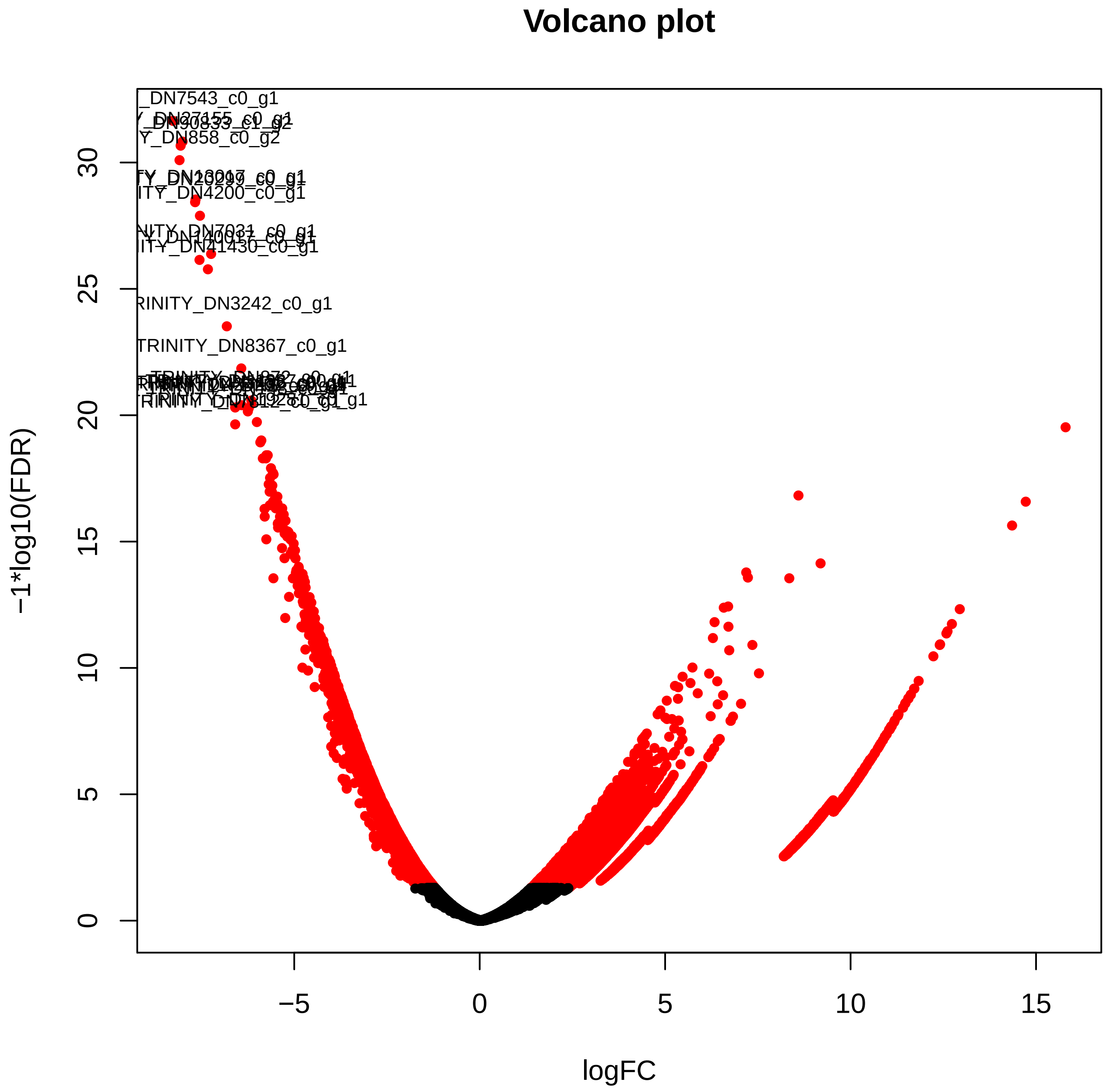

Volcano plot
logFC
−1*log10(FDR)
TRINITY_DN3242_c0_g1
TRINITY_DN8367_c0_g1
