# Supplementary figures and images for: Oogenesis and lipid metabolism in the deep-sea sponge Phakellia ventilabrum (Linnaeus, 1767)
Source: Sci Rep. 2022 Apr 15;12:6317. doi: 10.1038/s41598-022-10058-6 (PMC9012834; doi:10.1038/s41598-022-10058-6)

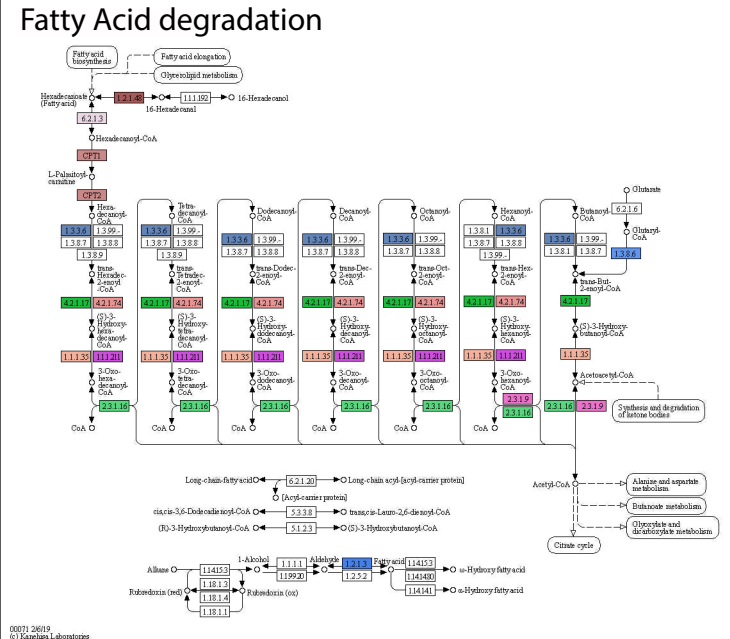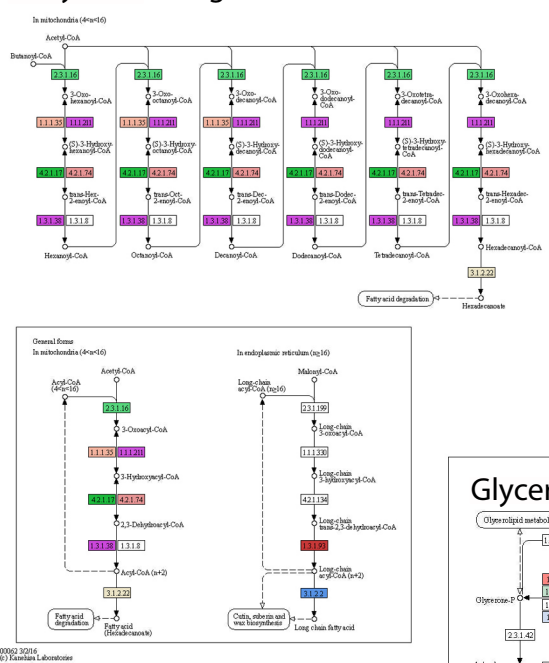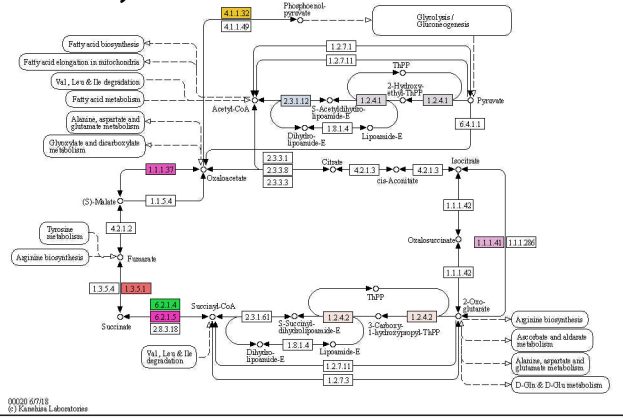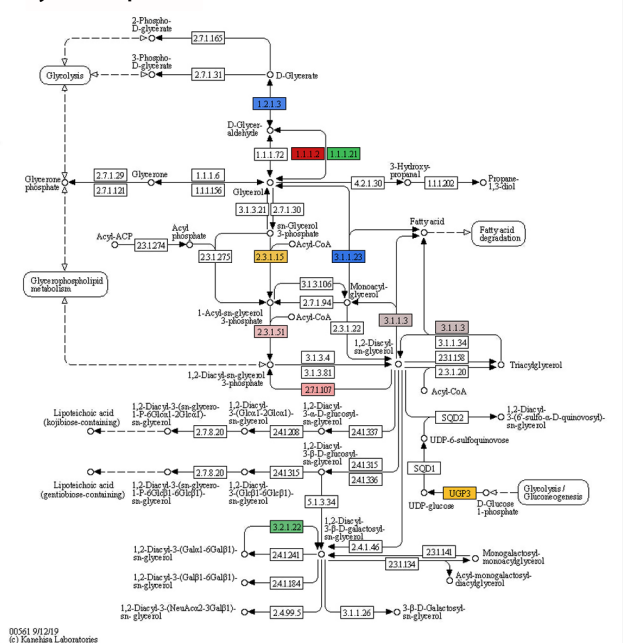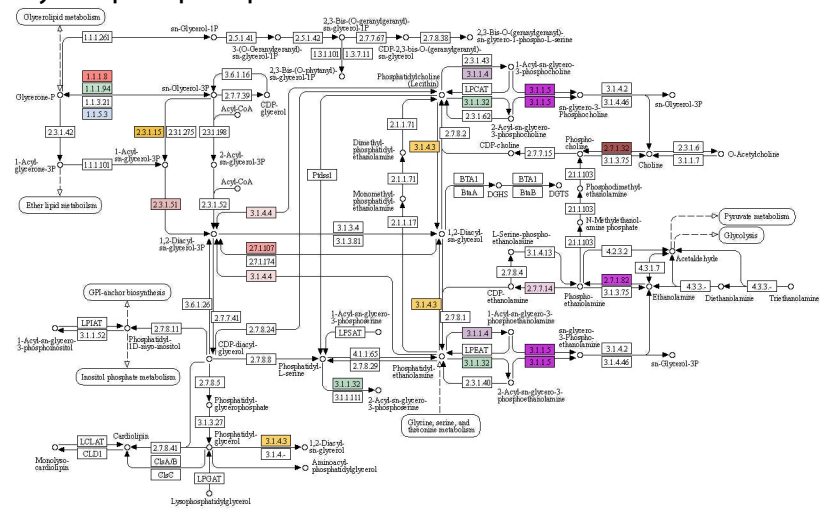

Supplement: Supplementary file 13 — Supplementary Figure S2. [file 41598_2022_10058_MOESM13_ESM.pdf]
